# Supplementary material for: Proteome-wide Mendelian randomization identifies causal links between blood proteins and severe COVID-19
Source: PLoS Genet. 2022 Mar 3;18(3):e1010042. doi: 10.1371/journal.pgen.1010042 (PMC8893330; doi:10.1371/journal.pgen.1010042)
Supplement: S1 Table — (DOCX) [file pgen.1010042.s001.docx]

# S1 Table. A breakdown of all studies from which inflammatory marker genome-wide association study (GWAS) data originated

| **First author** | **Date** | **PMID** | **Link** | **Summary statistics** | **Ancestry** | **Study name** | **Total N** | **Age** | **Number of males** | **Number of females** | **Number of markers** | **Platform** | **Normalization** | **Adjustment** |
| --- | --- | --- | --- | --- | --- | --- | --- | --- | --- | --- | --- | --- | --- | --- |
| Wood [1] | 2013 | 23696881 | <https://pubmed.ncbi.nlm.nih.gov/23696881/> | <https://www.ebi.ac.uk/gwas/publications/23696881> | European (Italy) | InCHIANTI | 1210 | Mean = 68.2, Range = 21-102 | 540 | 670 | 30 proteins selected (out of 93) | N/A | inverse-normalized 2 times | age, sex |
| Suhre [2] | 2017 | 28240269 | <https://pubmed.ncbi.nlm.nih.gov/28240269/> | <https://www.ebi.ac.uk/gwas/publications/28240269> | European (southern Germany) | KORA F4 | 997 | N/A | 483 | 514 | 1124 | SOMAscan | inverse-normalized probe levels | age, sex, BMI |
| Ahola-Olli  [3] | 2017 | 27989323 | <https://pubmed.ncbi.nlm.nih.gov/27989323/> | <https://www.ebi.ac.uk/gwas/publications/279893> | European (Finland) | Cardiovascular Risk in Young Finns Study & FINRISK | 840-8293 | N/A | N/A | N/A | 41 | Bio-Rad’s premixed Bio-Plex Pro Human Cytokine 27-plex Assay and 21-plex Assay, and Bio-Plex 200 reader | normalized with inverse transformation. | age, sex, BMI, 10 first PC |
| Folkersen  [4] | 2017 | 28369058 | <http://dx.plos.org/10.1371/journal.pgen.1006706> | <https://www.ebi.ac.uk/gwas/publications/28369058> | European (Finland, Sweden, France, Italy, Netherlands) | IMPROVE | 3394 | Mean = 64.5 (*) Range = 55-79 | N/A | N/A | 84 | OLINK | logarithmic | age, sex, recruitment center, protein analysis batch, smoking, diabetes and hypertension at baseline. |
| Sun  [5] | 2018 | 29875488 | <https://pubmed.ncbi.nlm.nih.gov/29875488/> | <https://www.ebi.ac.uk/gwas/publications/29875488> | European (England) | INTERVAL | 3301 | Mean = 43.7 | 1686 | 1615 | 3622 | SOMAscan | logarithmic | age, sex, duration between blood draw and processing, 3 first PC |
| Sliz  [6] | 2019 | 31217265 | <https://www.ncbi.nlm.nih.gov/pubmed/?term=31217265> | <https://www.ebi.ac.uk/gwas/publications/31217265>  Contacted corresponding author for full summary statistics | European (Finland) | Northern Finland Birth Cohort, meta-analysis for 10 markers (same as Ahola) | 5284 - 13577 | 31 (**) | 2543 (**) | 2741 (**) | 16 (**) | Bio-Rad’s premixed Bio-Plex Pro Human Cytokine 27-plex Assay and 21-plex Assay, and Bio-Plex 200 reader | rank-based inverse transformation | age, sex, BMI, 10 first PC |
| Bretherik  [7] | 2020 | 32628676 | <https://www.ncbi.nlm.nih.gov/pmc/articles/PMC7337286/> | <https://datashare.ed.ac.uk/handle/10283/3649> | European (Scottland, Croatia) | ORCADES and CROATIA-Vis | 971-993 (ORCADES), 887-899 (Croatia-VIS) | Range 16-100 (ORCADES), 18-93 (Croatia-VIS) | N/A | N/A | 249 | OLINK | rank-based, inverse-normal transformed | age, sex, genotyping array (ORCADES), proteomics plate, plate row, column, length of sample storage, sease (ORCADES), 10 first PC |
| Folkersen (“Scallop”)  [8] | 2020 | 33067605 | <https://www.nature.com/articles/s42255-020-00287-2#Abs1> | <https://zenodo.org/record/2615265#.YaD2uJDMLOQ> | European (except STABILITY) | IMPROVE, STANLEY, EpiHealth, PIVUS, ULSAM, INTERVAL, LifeLines-DEEP, NSPHS, STABILITY, Estonian BB, ORCADES, VIS, MPP-RES | up to 21,758 (mean 17747) | N/A | N/A | N/A | 90 | OLINK | rank-based, inverse-normal transformed | depending on cohort |
| Hoglund  [9] | 2019 | 31727947 | <https://www.nature.com/articles/s41598-019-53111-7> | <https://www.ebi.ac.uk/gwas/publications/31727947> | European (Northern Sweden Population) | Northern Swedish population health study (NSPHS) | 1069 | 14 to 94 years with a median of 52 year | N/A | 50.8% of the participants were females | 92 | OLINK | linear mixed-effects model | sex, age and batch effect |
| Hillary  [10] | 2019 | 31320639 | <https://www.nature.com/articles/s41467-019-11177-x> | <https://www.ebi.ac.uk/gwas/publications/31320639> | European (Scottland) | The Lothian Birth Cohort 1936 (LBC1936) | 750 | Approximately 70 years of age (mean age: 69.6 ± 0.8 years) | N/A | 49.8% female | 70 | OLINK | Linear regression | age, sex, four genetic principal components of ancestry |

(*) as reported in [11]

(**) for the Northern Finish Birth Cohort

PMID = PubMed ID

BMI = body mass index

EDTA = Ethylenediaminetetraacetic acid

PC = principal component

**References**:

[1] Wood, Andrew R., John R. B. Perry, Toshiko Tanaka, Dena G. Hernandez, Hou-Feng Zheng, David Melzer, J. Raphael Gibbs, et al. 2013. “Imputation of Variants from the 1000 Genomes Project Modestly Improves Known Associations and Can Identify Low-Frequency Variant-Phenotype Associations Undetected by HapMap Based Imputation.” *PloS One* 8 (5): e64343.

[2] Suhre, Karsten, Matthias Arnold, Aditya Mukund Bhagwat, Richard J. Cotton, Rudolf Engelke, Johannes Raffler, Hina Sarwath, et al. 2017. “Connecting Genetic Risk to Disease End Points through the Human Blood Plasma Proteome.” *Nature Communications* 8 (February): 14357.

[3] Ahola-Olli, Ari V., Peter Würtz, Aki S. Havulinna, Kristiina Aalto, Niina Pitkänen, Terho Lehtimäki, Mika Kähönen, et al. 2017. “Genome-Wide Association Study Identifies 27 Loci Influencing Concentrations of Circulating Cytokines and Growth Factors.” *American Journal of Human Genetics* 100: 40–50.

[4] Folkersen, Lasse, Stefan Gustafsson, Qin Wang, Daniel Hvidberg Hansen, Åsa K. Hedman, Andrew Schork, Karen Page, et al. 2020. “Genomic and Drug Target Evaluation of 90 Cardiovascular Proteins in 30,931 Individuals.” *Nature Metabolism* 2 (10): 1135–48.

[5] Sun, Benjamin B., Joseph C. Maranville, James E. Peters, David Stacey, James R. Staley, James Blackshaw, Stephen Burgess, et al. 2018. “Genomic Atlas of the Human Plasma Proteome.” *Nature* 558 (7708): 73–79.

[6] Sliz, Eeva, Marita Kalaoja, Ari Ahola-Olli, Olli Raitakari, Markus Perola, Veikko Salomaa, Terho Lehtimäki, et al. 2019. “Genome-Wide Association Study Identifies Seven Novel Loci Associating with Circulating Cytokines and Cell Adhesion Molecules in Finns.” *Journal of Medical Genetics* 56 (9): 607–16.

[7] Bretherick, Andrew D., Oriol Canela-Xandri, Peter K. Joshi, David W. Clark, Konrad Rawlik, Thibaud S. Boutin, Yanni Zeng, et al. 2020. “Linking Protein to Phenotype with Mendelian Randomization Detects 38 Proteins with Causal Roles in Human Diseases and Traits.” *PLoS Genetics* 16 (7): e1008785.

[8] Folkersen, Lasse, Stefan Gustafsson, Qin Wang, Daniel Hvidberg Hansen, Åsa K. Hedman, Andrew Schork, Karen Page, et al. 2020. “Genomic and Drug Target Evaluation of 90 Cardiovascular Proteins in 30,931 Individuals.” *Nature Metabolism* 2 (10): 1135–48.

[9] Höglund, Julia, Nima Rafati, Mathias Rask-Andersen, Stefan Enroth, Torgny Karlsson, Weronica E. Ek, and Åsa Johansson. 2019. “Improved Power and Precision with Whole Genome Sequencing Data in Genome-Wide Association Studies of Inflammatory Biomarkers.” *Scientific Reports* 9 (1): 16844.

[10] Hillary, Robert F., Daniel L. McCartney, Sarah E. Harris, Anna J. Stevenson, Anne Seeboth, Qian Zhang, David C. Liewald, et al. 2019. “Genome and Epigenome Wide Studies of Neurological Protein Biomarkers in the Lothian Birth Cohort 1936.” *Nature Communications* 10 (1): 3160.

[11] Strawbridge, Rona J., Anna Deleskog, Olga McLeod, Lasse Folkersen, Maryam Kavousi, Karl Gertow, Damiano Baldassarre et al. "A serum 25-hydroxyvitamin D concentration-associated genetic variant in DHCR7 interacts with type 2 diabetes status to influence subclinical atherosclerosis (measured by carotid intima–media thickness)." *Diabetologia* 57, no. 6 (2014): 1159-1172.
