# Supplementary material for: Proteome-wide Mendelian randomization identifies causal links between blood proteins and severe COVID-19
Source: PLoS Genet. 2022 Mar 3;18(3):e1010042. doi: 10.1371/journal.pgen.1010042 (PMC8893330; doi:10.1371/journal.pgen.1010042)
Supplement: S2 Table — (DOCX) [file pgen.1010042.s002.docx]

# S2 Table. Validation of our genetic instruments

| **Marker Name** | **F** | **I^2^** |
| --- | --- | --- |
| FAAH2_Sun | 52.281 | 0.892 |
| GCNT4_Sun | 50.828 | 0.884 |
| CD207_Sun | 95.114 | 0.969 |
| RAB14_Sun | 94.992 | 0.968 |
| C1GALT1C1_Sun | 42.255 | 0.918 |
| ABO_Sun | 443.924 | 0.993 |
| LCTL_Sun | 67.187 | 0.950 |
| SFTPD_Breth | 70.367 | 0.887 |
| SELL_Sun | 124.773 | 0.983 |
| SELE_Folk | 107.320 | 0.969 |
| KEL_Sun | 48.584 | 0.922 |
| SELE_Scal | 200.609 | 0.990 |
| SELE_Breth | 81.105 | 0.963 |
| ATP2A3_Sun | 25.550 | 0.787 |
| PECAM1_Scal | 109.033 | 0.976 |
| SELE_Sliz | 69.783 | 0.955 |
| sICAM1_Sliz | 42.314 | 0.867 |
| PECAM1_Folk | 136.403 | 0.997 |

This table displays details on the strength of our genetic instruments using an F-statistic (for IVW performance) and I-squared statistic (for MR-Egger).
