# Supplementary material for: Proteome-wide Mendelian randomization identifies causal links between blood proteins and severe COVID-19
Source: PLoS Genet. 2022 Mar 3;18(3):e1010042. doi: 10.1371/journal.pgen.1010042 (PMC8893330; doi:10.1371/journal.pgen.1010042)
Supplement: S3 Table — (DOCX) [file pgen.1010042.s003.docx]

# S3 Table. Reverse effect results from Mendelian randomization analyses

*This table details significant, false discovery rate-(FDR)-corrected Mendelian randomization results, using the Generalised Summary Data-based Mendelian randomization (GSMR) method, for reverse effects only. For all the analyses COVID-19 was the exposure of interest and blood protein level was the outcome of interest. No associations were found to be significant. The table presents the log odds statistics and corresponding standard error as well as odds ratios, 95% confidence intervals, and the FDR-adjusted Q values (p_FDR_ = 0.05).*

| **Exposure - Hospitalization as a Result of COVID-19** | | | | | | | | | |  |
| --- | --- | --- | --- | --- | --- | --- | --- | --- | --- | --- |
|  |  |  |  |  |  |  |  |  |  |  |
| **Protein (outcome)** | | **Beta** | **SE** | **p value** | **SNPs** | **OR** | **Lower 95% CI** | **Upper 95% CI** | ***Q*** |  |
| FAAH2_Sun | | 0.07 | 0.05 | 0.13 | 25 | 1.07 | 0.98 | 1.16 | 0.68 |  |
| KEL_Sun | | -0.07 | 0.05 | 0.12 | 24 | 0.93 | 0.84 | 1.02 | 0.68 |  |
| ATP2A3_Sun | | 0.08 | 0.04 | 0.08 | 26 | 1.08 | 0.99 | 1.17 | 0.68 |  |
| RAB14_Sun | | 0.05 | 0.05 | 0.23 | 25 | 1.06 | 0.97 | 1.15 | 0.69 |  |
| SFTPD_Breth | | 0.07 | 0.06 | 0.24 | 27 | 1.08 | 0.95 | 1.20 | 0.69 |  |
| PECAM1_Scal | | 0.02 | 0.02 | 0.26 | 25 | 1.02 | 0.99 | 1.05 | 0.69 |  |
| CD207_Sun | | 0.04 | 0.05 | 0.33 | 25 | 1.05 | 0.96 | 1.13 | 0.69 |  |
| ABO_Sun | | 0.04 | 0.05 | 0.34 | 25 | 1.04 | 0.95 | 1.13 | 0.69 |  |
| C1GALT1C1_Sun | | -0.04 | 0.05 | 0.41 | 25 | 0.96 | 0.87 | 1.05 | 0.74 |  |
| LCTL_Sun | | 0.02 | 0.05 | 0.60 | 25 | 1.02 | 0.94 | 1.11 | 0.74 |  |
| SELE_Scal | | -0.01 | 0.02 | 0.57 | 25 | 0.99 | 0.96 | 1.02 | 0.74 |  |
| SELE_Breth | | 0.04 | 0.06 | 0.48 | 28 | 1.04 | 0.93 | 1.15 | 0.74 |  |
| GCNT4_Sun | | 0.00 | 0.05 | 0.97 | 25 | 1.00 | 0.91 | 1.09 | 1.00 |  |
| SELL_Sun | | 0.00 | 0.05 | 1.00 | 25 | 1.00 | 0.91 | 1.09 | 1.00 |  |
| SELE_Folk | | 0.00 | 0.06 | 0.95 | 17 | 1.00 | 0.89 | 1.11 | 1.00 |  |
| **Outcome - Hospitalization as a Result of COVID-19** | | | | | | | | | |  |
|  |  |  |  |  |  |  |  |  |  |  |
| **Protein (exposure)** | | **Beta** | **SE** | **p value** | **SNPs** | **OR** | **Lower 95% CI** | **Upper 95% CI** | ***Q*** |  |
| MIP1b_Ahol | | -0.07 | 0.02 | 0.00 | 87 | 0.93 | 0.90 | 0.97 | 0.07 |  |
|  |  |  |  |  |  |  |  |  |  |  |
| **Exposure - Respiratory support/death as a result of COVID-19** | | | | | | | | | |  |
|  |  |  |  |  |  |  |  |  |  |  |
| **Protein (outcome)** | | **Beta** | **SE** | **p value** | **SNPs** | **OR** | **Lower 95% CI** | **Upper 95% CI** | ***Q*** |  |
| C1GALT1C1_Sun | | -0.06 | 0.03 | 0.03 | 31 | 0.94 | 0.88 | 1.00 | 0.41 |  |
| sICAM1_Sliz | | -0.04 | 0.02 | 0.06 | 32 | 0.96 | 0.92 | 1.00 | 0.41 |  |
| ABO_Sun | | -0.03 | 0.03 | 0.23 | 31 | 0.97 | 0.91 | 1.02 | 0.64 |  |
| SELL_Sun | | 0.04 | 0.03 | 0.22 | 31 | 1.04 | 0.98 | 1.09 | 0.64 |  |
| SELE_Scal | | -0.01 | 0.01 | 0.19 | 32 | 0.99 | 0.96 | 1.01 | 0.64 |  |
| RAB14_Sun | | 0.03 | 0.03 | 0.30 | 30 | 1.03 | 0.97 | 1.09 | 0.71 |  |
| GCNT4_Sun | | -0.02 | 0.03 | 0.49 | 31 | 0.98 | 0.92 | 1.04 | 0.74 |  |
| SELE_Sliz | | -0.01 | 0.02 | 0.58 | 32 | 0.99 | 0.94 | 1.03 | 0.74 |  |
| SELE_Breth | | -0.03 | 0.04 | 0.43 | 32 | 0.97 | 0.89 | 1.05 | 0.74 |  |
| PECAM1_Scal | | 0.01 | 0.01 | 0.57 | 32 | 1.01 | 0.98 | 1.03 | 0.74 |  |
| CD207_Sun | | 0.01 | 0.03 | 0.75 | 31 | 1.01 | 0.95 | 1.07 | 0.76 |  |
| SELE_Folk | | -0.01 | 0.04 | 0.74 | 19 | 0.99 | 0.92 | 1.06 | 0.76 |  |
| PECAM1_Folk | | 0.01 | 0.04 | 0.76 | 19 | 1.01 | 0.94 | 1.08 | 0.76 |  |
| **Outcome - Respiratory support/death as a result of COVID-19** | | | | | | | | | |  |
|  |  |  |  |  |  |  |  |  |  |  |
| **Protein (exposure)** | | **Beta** | **SE** | **p value** | **SNPs** | **OR** | **Lower 95% CI** | **Upper 95% CI** | ***Q*** |  |
| *N/A* | | *N/A* | *N/A* | *N/A* | *N/A* | *N/A* | *N/A* | *N/A* | *N/A* |  |

*Note: SE = standard error, SNPs = number of single nucleotide polymorphisms in common between the exposure and outcome; OR = odds ratio, CI = confidence interval, Q = false discovery rate-adjusted Q value; ABO = ABO system transferase; ATP2A3 = ATPase Sarcoplasmic/Endoplasmic Reticulum Ca2+ Transporting 3; C1GALT1C1 = C1GALT1 specific chaperone 1; CD207 = langerin; FAAH2 = Fatty Acid Amide Hydrolase 2; GCNT4 = glucosaminyl (N-Acetyl) transferase 4; KEL = Kell Metallo-Endopeptidase (Kell Blood Group); LCTL = Lactase-like protein; MIP1b = macrophage inflammatory protein; NEP = neprilysin; PECAM1 = platelet endothelial cell adhesion molecule; RAB14 = ras-related protein rab-14; SELE = E-selectin; SELL =  L-selectin; SFTPD = Surfactant Protein D; sICAM1= Soluble intercellular adhesion molecule-1.*

# 
