# Supplementary material for: Proteome-wide Mendelian randomization identifies causal links between blood proteins and severe COVID-19
Source: PLoS Genet. 2022 Mar 3;18(3):e1010042. doi: 10.1371/journal.pgen.1010042 (PMC8893330; doi:10.1371/journal.pgen.1010042)
Supplement: S4 Table — (DOCX) [file pgen.1010042.s004.docx]

# S4 Table. Results from sensitivity analyses for all markers and risk hospitalization as a result of COVID-19

| **Exposure** | **Outcome** | **Method** | **SNPs** | **BETA** | **SE** | **P** |
| --- | --- | --- | --- | --- | --- | --- |
| FAAH2_Sun | Hospitalized_Covid | Maximum likelihood | 13 | 0.174 | 0.033 | 0.000 |
| FAAH2_Sun | Hospitalized_Covid | MR Egger | 13 | 0.303 | 0.086 | 0.005 |
| FAAH2_Sun | Hospitalized_Covid | Weighted median | 13 | 0.233 | 0.043 | 0.000 |
| FAAH2_Sun | Hospitalized_Covid | Inverse variance weighted | 13 | 0.170 | 0.038 | 0.000 |
| FAAH2_Sun | Hospitalized_Covid | IVW radial | 13 | 0.171 | 0.038 | 0.000 |
| FAAH2_Sun | Hospitalized_Covid | Inverse variance weighted (multiplicative random effects) | 13 | 0.170 | 0.038 | 0.000 |
| FAAH2_Sun | Hospitalized_Covid | Inverse variance weighted (fixed effects) | 13 | 0.170 | 0.032 | 0.000 |
| FAAH2_Sun | Hospitalized_Covid | Weighted mode | 13 | 0.235 | 0.047 | 0.000 |
|  |  |  |  |  |  |  |
| GCNT4_Sun | Hospitalized_Covid | Maximum likelihood | 18 | 0.150 | 0.028 | 0.000 |
| GCNT4_Sun | Hospitalized_Covid | MR Egger | 18 | 0.258 | 0.089 | 0.011 |
| GCNT4_Sun | Hospitalized_Covid | Weighted median | 18 | 0.181 | 0.043 | 0.000 |
| GCNT4_Sun | Hospitalized_Covid | Inverse variance weighted | 18 | 0.148 | 0.036 | 0.000 |
| GCNT4_Sun | Hospitalized_Covid | IVW radial | 18 | 0.149 | 0.036 | 0.000 |
| GCNT4_Sun | Hospitalized_Covid | Inverse variance weighted (multiplicative random effects) | 18 | 0.148 | 0.036 | 0.000 |
| GCNT4_Sun | Hospitalized_Covid | Inverse variance weighted (fixed effects) | 18 | 0.148 | 0.027 | 0.000 |
| GCNT4_Sun | Hospitalized_Covid | Weighted mode | 18 | 0.206 | 0.039 | 0.000 |
|  |  |  |  |  |  |  |
| CD207_Sun | Hospitalized_Covid | Maximum likelihood | 23 | 0.112 | 0.020 | 0.000 |
| CD207_Sun | Hospitalized_Covid | MR Egger | 23 | 0.147 | 0.035 | 0.000 |
| CD207_Sun | Hospitalized_Covid | Weighted median | 23 | 0.149 | 0.028 | 0.000 |
| CD207_Sun | Hospitalized_Covid | Inverse variance weighted | 23 | 0.111 | 0.019 | 0.000 |
| CD207_Sun | Hospitalized_Covid | IVW radial | 23 | 0.111 | 0.018 | 0.000 |
| CD207_Sun | Hospitalized_Covid | Inverse variance weighted (multiplicative random effects) | 23 | 0.111 | 0.018 | 0.000 |
| CD207_Sun | Hospitalized_Covid | Inverse variance weighted (fixed effects) | 23 | 0.111 | 0.019 | 0.000 |
| CD207_Sun | Hospitalized_Covid | Weighted mode | 23 | 0.150 | 0.030 | 0.000 |
|  |  |  |  |  |  |  |
| RAB14_Sun | Hospitalized_Covid | Maximum likelihood | 24 | 0.104 | 0.019 | 0.000 |
| RAB14_Sun | Hospitalized_Covid | MR Egger | 24 | 0.142 | 0.034 | 0.000 |
| RAB14_Sun | Hospitalized_Covid | Weighted median | 24 | 0.124 | 0.025 | 0.000 |
| RAB14_Sun | Hospitalized_Covid | Inverse variance weighted | 24 | 0.103 | 0.019 | 0.000 |
| RAB14_Sun | Hospitalized_Covid | IVW radial | 24 | 0.103 | 0.015 | 0.000 |
| RAB14_Sun | Hospitalized_Covid | Inverse variance weighted (multiplicative random effects) | 24 | 0.103 | 0.015 | 0.000 |
| RAB14_Sun | Hospitalized_Covid | Inverse variance weighted (fixed effects) | 24 | 0.103 | 0.019 | 0.000 |
| RAB14_Sun | Hospitalized_Covid | Weighted mode | 24 | 0.121 | 0.025 | 0.000 |
|  |  |  |  |  |  |  |
| C1GALT1C1_Sun | Hospitalized_Covid | Maximum likelihood | 23 | 0.089 | 0.027 | 0.001 |
| C1GALT1C1_Sun | Hospitalized_Covid | MR Egger | 23 | 0.155 | 0.061 | 0.020 |
| C1GALT1C1_Sun | Hospitalized_Covid | Weighted median | 23 | 0.148 | 0.039 | 0.000 |
| C1GALT1C1_Sun | Hospitalized_Covid | Inverse variance weighted | 23 | 0.086 | 0.032 | 0.007 |
| C1GALT1C1_Sun | Hospitalized_Covid | IVW radial | 23 | 0.086 | 0.032 | 0.007 |
| C1GALT1C1_Sun | Hospitalized_Covid | Inverse variance weighted (multiplicative random effects) | 23 | 0.086 | 0.032 | 0.007 |
| C1GALT1C1_Sun | Hospitalized_Covid | Inverse variance weighted (fixed effects) | 23 | 0.086 | 0.026 | 0.001 |
| C1GALT1C1_Sun | Hospitalized_Covid | Weighted mode | 23 | 0.184 | 0.042 | 0.000 |
|  |  |  |  |  |  |  |
| ABO_Sun | Hospitalized_Covid | Maximum likelihood | 17 | 0.081 | 0.014 | 0.000 |
| ABO_Sun | Hospitalized_Covid | MR Egger | 17 | 0.105 | 0.025 | 0.001 |
| ABO_Sun | Hospitalized_Covid | Weighted median | 17 | 0.096 | 0.017 | 0.000 |
| ABO_Sun | Hospitalized_Covid | Inverse variance weighted | 17 | 0.081 | 0.014 | 0.000 |
| ABO_Sun | Hospitalized_Covid | IVW radial | 17 | 0.081 | 0.014 | 0.000 |
| ABO_Sun | Hospitalized_Covid | Inverse variance weighted (multiplicative random effects) | 17 | 0.081 | 0.014 | 0.000 |
| ABO_Sun | Hospitalized_Covid | Inverse variance weighted (fixed effects) | 17 | 0.081 | 0.014 | 0.000 |
| ABO_Sun | Hospitalized_Covid | Weighted mode | 17 | 0.092 | 0.017 | 0.000 |
|  |  |  |  |  |  |  |
| LCTL_Sun | Hospitalized_Covid | Maximum likelihood | 39 | -0.077 | 0.016 | 0.000 |
| LCTL_Sun | Hospitalized_Covid | MR Egger | 39 | -0.091 | 0.031 | 0.005 |
| LCTL_Sun | Hospitalized_Covid | Weighted median | 39 | -0.082 | 0.025 | 0.001 |
| LCTL_Sun | Hospitalized_Covid | Inverse variance weighted | 39 | -0.077 | 0.016 | 0.000 |
| LCTL_Sun | Hospitalized_Covid | IVW radial | 39 | -0.077 | 0.012 | 0.000 |
| LCTL_Sun | Hospitalized_Covid | Inverse variance weighted (multiplicative random effects) | 39 | -0.077 | 0.012 | 0.000 |
| LCTL_Sun | Hospitalized_Covid | Inverse variance weighted (fixed effects) | 39 | -0.077 | 0.016 | 0.000 |
| LCTL_Sun | Hospitalized_Covid | Weighted mode | 39 | -0.087 | 0.028 | 0.004 |
|  |  |  |  |  |  |  |
| SFTPD_Breth | Hospitalized_Covid | Maximum likelihood | 6 | -0.097 | 0.027 | 0.000 |
| SFTPD_Breth | Hospitalized_Covid | MR Egger | 6 | -0.086 | 0.151 | 0.599 |
| SFTPD_Breth | Hospitalized_Covid | Weighted median | 6 | -0.090 | 0.041 | 0.026 |
| SFTPD_Breth | Hospitalized_Covid | Inverse variance weighted | 6 | -0.095 | 0.042 | 0.024 |
| SFTPD_Breth | Hospitalized_Covid | IVW radial | 6 | -0.095 | 0.042 | 0.024 |
| SFTPD_Breth | Hospitalized_Covid | Inverse variance weighted (multiplicative random effects) | 6 | -0.095 | 0.042 | 0.024 |
| SFTPD_Breth | Hospitalized_Covid | Inverse variance weighted (fixed effects) | 6 | -0.095 | 0.026 | 0.000 |
| SFTPD_Breth | Hospitalized_Covid | Weighted mode | 6 | -0.115 | 0.038 | 0.029 |
|  |  |  |  |  |  |  |
| SELL_Sun | Hospitalized_Covid | Maximum likelihood | 24 | -0.091 | 0.018 | 0.000 |
| SELL_Sun | Hospitalized_Covid | MR Egger | 24 | -0.112 | 0.028 | 0.001 |
| SELL_Sun | Hospitalized_Covid | Weighted median | 24 | -0.103 | 0.023 | 0.000 |
| SELL_Sun | Hospitalized_Covid | Inverse variance weighted | 24 | -0.090 | 0.018 | 0.000 |
| SELL_Sun | Hospitalized_Covid | IVW radial | 24 | -0.090 | 0.016 | 0.000 |
| SELL_Sun | Hospitalized_Covid | Inverse variance weighted (multiplicative random effects) | 24 | -0.090 | 0.016 | 0.000 |
| SELL_Sun | Hospitalized_Covid | Inverse variance weighted (fixed effects) | 24 | -0.090 | 0.018 | 0.000 |
| SELL_Sun | Hospitalized_Covid | Weighted mode | 24 | -0.106 | 0.022 | 0.000 |
|  |  |  |  |  |  |  |
| SELE_Folk | Hospitalized_Covid | Maximum likelihood | 16 | -0.113 | 0.021 | 0.000 |
| SELE_Folk | Hospitalized_Covid | MR Egger | 16 | -0.141 | 0.046 | 0.009 |
| SELE_Folk | Hospitalized_Covid | Weighted median | 16 | -0.141 | 0.027 | 0.000 |
| SELE_Folk | Hospitalized_Covid | Inverse variance weighted | 16 | -0.109 | 0.024 | 0.000 |
| SELE_Folk | Hospitalized_Covid | IVW radial | 16 | -0.110 | 0.023 | 0.000 |
| SELE_Folk | Hospitalized_Covid | Inverse variance weighted (multiplicative random effects) | 16 | -0.109 | 0.024 | 0.000 |
| SELE_Folk | Hospitalized_Covid | Inverse variance weighted (fixed effects) | 16 | -0.109 | 0.020 | 0.000 |
| SELE_Folk | Hospitalized_Covid | Weighted mode | 16 | -0.144 | 0.026 | 0.000 |
|  |  |  |  |  |  |  |
| KEL_Sun | Hospitalized_Covid | Maximum likelihood | 17 | -0.119 | 0.030 | 0.000 |
| KEL_Sun | Hospitalized_Covid | MR Egger | 17 | -0.219 | 0.068 | 0.005 |
| KEL_Sun | Hospitalized_Covid | Weighted median | 17 | -0.175 | 0.041 | 0.000 |
| KEL_Sun | Hospitalized_Covid | Inverse variance weighted | 17 | -0.118 | 0.035 | 0.001 |
| KEL_Sun | Hospitalized_Covid | IVW radial | 17 | -0.118 | 0.035 | 0.001 |
| KEL_Sun | Hospitalized_Covid | Inverse variance weighted (multiplicative random effects) | 17 | -0.118 | 0.035 | 0.001 |
| KEL_Sun | Hospitalized_Covid | Inverse variance weighted (fixed effects) | 17 | -0.118 | 0.029 | 0.000 |
| KEL_Sun | Hospitalized_Covid | Weighted mode | 17 | -0.195 | 0.043 | 0.000 |
|  |  |  |  |  |  |  |
| SELE_Scal | Hospitalized_Covid | Maximum likelihood | 50 | -0.122 | 0.022 | 0.000 |
| SELE_Scal | Hospitalized_Covid | MR Egger | 50 | -0.139 | 0.035 | 0.000 |
| SELE_Scal | Hospitalized_Covid | Weighted median | 50 | -0.149 | 0.030 | 0.000 |
| SELE_Scal | Hospitalized_Covid | Inverse variance weighted | 50 | -0.122 | 0.024 | 0.000 |
| SELE_Scal | Hospitalized_Covid | IVW radial | 50 | -0.122 | 0.024 | 0.000 |
| SELE_Scal | Hospitalized_Covid | Inverse variance weighted (multiplicative random effects) | 50 | -0.122 | 0.024 | 0.000 |
| SELE_Scal | Hospitalized_Covid | Inverse variance weighted (fixed effects) | 50 | -0.122 | 0.022 | 0.000 |
| SELE_Scal | Hospitalized_Covid | Weighted mode | 50 | -0.149 | 0.026 | 0.000 |
|  |  |  |  |  |  |  |
| SELE_Breth | Hospitalized_Covid | Maximum likelihood | 6 | -0.134 | 0.030 | 0.000 |
| SELE_Breth | Hospitalized_Covid | MR Egger | 6 | -0.219 | 0.082 | 0.056 |
| SELE_Breth | Hospitalized_Covid | Weighted median | 6 | -0.168 | 0.035 | 0.000 |
| SELE_Breth | Hospitalized_Covid | Inverse variance weighted | 6 | -0.129 | 0.046 | 0.005 |
| SELE_Breth | Hospitalized_Covid | IVW radial | 6 | -0.130 | 0.045 | 0.004 |
| SELE_Breth | Hospitalized_Covid | Inverse variance weighted (multiplicative random effects) | 6 | -0.129 | 0.046 | 0.005 |
| SELE_Breth | Hospitalized_Covid | Inverse variance weighted (fixed effects) | 6 | -0.129 | 0.029 | 0.000 |
| SELE_Breth | Hospitalized_Covid | Weighted mode | 6 | -0.165 | 0.037 | 0.007 |
|  |  |  |  |  |  |  |
| ATP2A3_Sun | Hospitalized_Covid | Maximum likelihood | 16 | -0.155 | 0.040 | 0.000 |
| ATP2A3_Sun | Hospitalized_Covid | MR Egger | 16 | -0.045 | 0.092 | 0.635 |
| ATP2A3_Sun | Hospitalized_Covid | Weighted median | 16 | -0.140 | 0.055 | 0.011 |
| ATP2A3_Sun | Hospitalized_Covid | Inverse variance weighted | 16 | -0.155 | 0.039 | 0.000 |
| ATP2A3_Sun | Hospitalized_Covid | IVW radial | 16 | -0.155 | 0.037 | 0.000 |
| ATP2A3_Sun | Hospitalized_Covid | Inverse variance weighted (multiplicative random effects) | 16 | -0.155 | 0.036 | 0.000 |
| ATP2A3_Sun | Hospitalized_Covid | Inverse variance weighted (fixed effects) | 16 | -0.155 | 0.039 | 0.000 |
| ATP2A3_Sun | Hospitalized_Covid | Weighted mode | 16 | -0.178 | 0.092 | 0.072 |
|  |  |  |  |  |  |  |
| PECAM1_Scal | Hospitalized_Covid | Maximum likelihood | 29 | -0.229 | 0.036 | 0.000 |
| PECAM1_Scal | Hospitalized_Covid | MR Egger | 29 | -0.222 | 0.057 | 0.001 |
| PECAM1_Scal | Hospitalized_Covid | Weighted median | 29 | -0.256 | 0.048 | 0.000 |
| PECAM1_Scal | Hospitalized_Covid | Inverse variance weighted | 29 | -0.230 | 0.036 | 0.000 |
| PECAM1_Scal | Hospitalized_Covid | IVW radial | 29 | -0.230 | 0.033 | 0.000 |
| PECAM1_Scal | Hospitalized_Covid | Inverse variance weighted (multiplicative random effects) | 29 | -0.230 | 0.033 | 0.000 |
| PECAM1_Scal | Hospitalized_Covid | Inverse variance weighted (fixed effects) | 29 | -0.230 | 0.036 | 0.000 |
| PECAM1_Scal | Hospitalized_Covid | Weighted mode | 29 | -0.252 | 0.044 | 0.000 |

Number of SNPS = nsnp / Beta = b / Standard Error = se / P-value = pval

*ABO = ABO system transferase; ATP2A3 = ATPase Sarcoplasmic/Endoplasmic Reticulum Ca2+ Transporting 3; C1GALT1C1 = C1GALT1 specific chaperone 1; CD207 = langerin; FAAH2 = Fatty Acid Amide Hydrolase 2; GCNT4 = glucosaminyl (N-Acetyl) transferase 4; KEL = Kell Metallo-Endopeptidase (Kell Blood Group); LCTL = Lactase-like protein; PECAM1 = platelet endothelial cell adhesion molecule; RAB14 = ras-related protein rab-14; SELE = E-selectin; SELL =  L-selectin; SFTPD = Surfactant Protein D.*
