# Supplementary material for: Proteome-wide Mendelian randomization identifies causal links between blood proteins and severe COVID-19
Source: PLoS Genet. 2022 Mar 3;18(3):e1010042. doi: 10.1371/journal.pgen.1010042 (PMC8893330; doi:10.1371/journal.pgen.1010042)
Supplement: S5 Table — (DOCX) [file pgen.1010042.s005.docx]

# S5 Table. Results from sensitivity analyses for all markers and respiratory support/death as a result of COVID-19

| **Exposure** | **Outcome** | **Method** | **SNPs** | **BETA** | **SE** | **P** |
| --- | --- | --- | --- | --- | --- | --- |
| GCNT4_Sun | Respiratory support or death | Maximum likelihood | 16 | 0.313 | 0.047 | 0.000 |
| GCNT4_Sun | Respiratory support or death | MR Egger | 16 | 0.188 | 0.128 | 0.166 |
| GCNT4_Sun | Respiratory support or death | Simple median | 16 | 0.290 | 0.074 | 0.000 |
| GCNT4_Sun | Respiratory support or death | Weighted median | 16 | 0.322 | 0.062 | 0.000 |
| GCNT4_Sun | Respiratory support or death | Inverse variance weighted | 16 | 0.302 | 0.053 | 0.000 |
| GCNT4_Sun | Respiratory support or death | IVW radial | 16 | 0.302 | 0.053 | 0.000 |
| GCNT4_Sun | Respiratory support or death | Inverse variance weighted (multiplicative random effects) | 16 | 0.302 | 0.053 | 0.000 |
| GCNT4_Sun | Respiratory support or death | Inverse variance weighted (fixed effects) | 16 | 0.302 | 0.044 | 0.000 |
| GCNT4_Sun | Respiratory support or death | Simple mode | 16 | 0.317 | 0.110 | 0.011 |
| GCNT4_Sun | Respiratory support or death | Weighted mode | 16 | 0.328 | 0.064 | 0.000 |
|  |  |  |  |  |  |  |
| RAB14_Sun | Respiratory support or death | Maximum likelihood | 27 | 0.204 | 0.030 | 0.000 |
| RAB14_Sun | Respiratory support or death | MR Egger | 27 | 0.230 | 0.080 | 0.008 |
| RAB14_Sun | Respiratory support or death | Simple median | 27 | 0.199 | 0.058 | 0.001 |
| RAB14_Sun | Respiratory support or death | Weighted median | 27 | 0.205 | 0.041 | 0.000 |
| RAB14_Sun | Respiratory support or death | Inverse variance weighted | 27 | 0.201 | 0.042 | 0.000 |
| RAB14_Sun | Respiratory support or death | IVW radial | 27 | 0.201 | 0.042 | 0.000 |
| RAB14_Sun | Respiratory support or death | Inverse variance weighted (multiplicative random effects) | 27 | 0.201 | 0.042 | 0.000 |
| RAB14_Sun | Respiratory support or death | Inverse variance weighted (fixed effects) | 27 | 0.201 | 0.029 | 0.000 |
| RAB14_Sun | Respiratory support or death | Simple mode | 27 | 0.145 | 0.093 | 0.132 |
| RAB14_Sun | Respiratory support or death | Weighted mode | 27 | 0.203 | 0.038 | 0.000 |
|  |  |  |  |  |  |  |
| C1GALT1C1_Sun | Respiratory support or death | Maximum likelihood | 25 | 0.149 | 0.041 | 0.000 |
| C1GALT1C1_Sun | Respiratory support or death | MR Egger | 25 | 0.290 | 0.086 | 0.003 |
| C1GALT1C1_Sun | Respiratory support or death | Simple median | 25 | 0.064 | 0.074 | 0.388 |
| C1GALT1C1_Sun | Respiratory support or death | Weighted median | 25 | 0.233 | 0.063 | 0.000 |
| C1GALT1C1_Sun | Respiratory support or death | Inverse variance weighted | 25 | 0.144 | 0.049 | 0.003 |
| C1GALT1C1_Sun | Respiratory support or death | IVW radial | 25 | 0.144 | 0.049 | 0.003 |
| C1GALT1C1_Sun | Respiratory support or death | Inverse variance weighted (multiplicative random effects) | 25 | 0.144 | 0.049 | 0.003 |
| C1GALT1C1_Sun | Respiratory support or death | Inverse variance weighted (fixed effects) | 25 | 0.144 | 0.040 | 0.000 |
| C1GALT1C1_Sun | Respiratory support or death | Simple mode | 25 | 0.285 | 0.150 | 0.070 |
| C1GALT1C1_Sun | Respiratory support or death | Weighted mode | 25 | 0.294 | 0.070 | 0.000 |
|  |  |  |  |  |  |  |
| CD207_Sun | Respiratory support or death | Maximum likelihood | 23 | 0.168 | 0.033 | 0.000 |
| CD207_Sun | Respiratory support or death | MR Egger | 23 | 0.147 | 0.061 | 0.024 |
| CD207_Sun | Respiratory support or death | Simple median | 23 | 0.140 | 0.060 | 0.020 |
| CD207_Sun | Respiratory support or death | Weighted median | 23 | 0.187 | 0.043 | 0.000 |
| CD207_Sun | Respiratory support or death | Inverse variance weighted | 23 | 0.165 | 0.034 | 0.000 |
| CD207_Sun | Respiratory support or death | IVW radial | 23 | 0.165 | 0.034 | 0.000 |
| CD207_Sun | Respiratory support or death | Inverse variance weighted (multiplicative random effects) | 23 | 0.165 | 0.034 | 0.000 |
| CD207_Sun | Respiratory support or death | Inverse variance weighted (fixed effects) | 23 | 0.165 | 0.032 | 0.000 |
| CD207_Sun | Respiratory support or death | Simple mode | 23 | 0.137 | 0.097 | 0.172 |
| CD207_Sun | Respiratory support or death | Weighted mode | 23 | 0.186 | 0.044 | 0.000 |
|  |  |  |  |  |  |  |
| ABO_Sun | Respiratory support or death | Maximum likelihood | 29 | 0.119 | 0.021 | 0.000 |
| ABO_Sun | Respiratory support or death | MR Egger | 29 | 0.120 | 0.036 | 0.003 |
| ABO_Sun | Respiratory support or death | Simple median | 29 | 0.063 | 0.044 | 0.152 |
| ABO_Sun | Respiratory support or death | Weighted median | 29 | 0.120 | 0.028 | 0.000 |
| ABO_Sun | Respiratory support or death | Inverse variance weighted | 29 | 0.118 | 0.024 | 0.000 |
| ABO_Sun | Respiratory support or death | IVW radial | 29 | 0.118 | 0.024 | 0.000 |
| ABO_Sun | Respiratory support or death | Inverse variance weighted (multiplicative random effects) | 29 | 0.118 | 0.024 | 0.000 |
| ABO_Sun | Respiratory support or death | Inverse variance weighted (fixed effects) | 29 | 0.118 | 0.021 | 0.000 |
| ABO_Sun | Respiratory support or death | Simple mode | 29 | 0.048 | 0.067 | 0.481 |
| ABO_Sun | Respiratory support or death | Weighted mode | 29 | 0.121 | 0.026 | 0.000 |
|  |  |  |  |  |  |  |
| SELE_Sliz | Respiratory support or death | Maximum likelihood | 64 | -0.108 | 0.026 | 0.000 |
| SELE_Sliz | Respiratory support or death | MR Egger | 64 | -0.168 | 0.051 | 0.002 |
| SELE_Sliz | Respiratory support or death | Simple median | 64 | -0.082 | 0.047 | 0.084 |
| SELE_Sliz | Respiratory support or death | Weighted median | 64 | -0.170 | 0.041 | 0.000 |
| SELE_Sliz | Respiratory support or death | Inverse variance weighted | 64 | -0.108 | 0.029 | 0.000 |
| SELE_Sliz | Respiratory support or death | IVW radial | 64 | -0.108 | 0.029 | 0.000 |
| SELE_Sliz | Respiratory support or death | Inverse variance weighted (multiplicative random effects) | 64 | -0.108 | 0.029 | 0.000 |
| SELE_Sliz | Respiratory support or death | Inverse variance weighted (fixed effects) | 64 | -0.108 | 0.025 | 0.000 |
| SELE_Sliz | Respiratory support or death | Simple mode | 64 | -0.168 | 0.095 | 0.082 |
| SELE_Sliz | Respiratory support or death | Weighted mode | 64 | -0.172 | 0.040 | 0.000 |
|  |  |  |  |  |  |  |
| SELL_Sun | Respiratory support or death | Maximum likelihood | 24 | -0.127 | 0.029 | 0.000 |
| SELL_Sun | Respiratory support or death | MR Egger | 24 | -0.157 | 0.046 | 0.002 |
| SELL_Sun | Respiratory support or death | Simple median | 24 | -0.136 | 0.061 | 0.025 |
| SELL_Sun | Respiratory support or death | Weighted median | 24 | -0.163 | 0.035 | 0.000 |
| SELL_Sun | Respiratory support or death | Inverse variance weighted | 24 | -0.126 | 0.029 | 0.000 |
| SELL_Sun | Respiratory support or death | IVW radial | 24 | -0.127 | 0.029 | 0.000 |
| SELL_Sun | Respiratory support or death | Inverse variance weighted (multiplicative random effects) | 24 | -0.126 | 0.029 | 0.000 |
| SELL_Sun | Respiratory support or death | Inverse variance weighted (fixed effects) | 24 | -0.126 | 0.028 | 0.000 |
| SELL_Sun | Respiratory support or death | Simple mode | 24 | -0.205 | 0.110 | 0.076 |
| SELL_Sun | Respiratory support or death | Weighted mode | 24 | -0.168 | 0.034 | 0.000 |
|  |  |  |  |  |  |  |
| SELE_Scal | Respiratory support or death | Maximum likelihood | 6 | -0.242 | 0.047 | 0.000 |
| SELE_Scal | Respiratory support or death | MR Egger | 6 | -0.242 | 0.060 | 0.016 |
| SELE_Scal | Respiratory support or death | Simple median | 6 | -0.156 | 0.243 | 0.520 |
| SELE_Scal | Respiratory support or death | Weighted median | 6 | -0.232 | 0.049 | 0.000 |
| SELE_Scal | Respiratory support or death | Inverse variance weighted | 6 | -0.242 | 0.047 | 0.000 |
| SELE_Scal | Respiratory support or death | IVW radial | 6 | -0.242 | 0.031 | 0.000 |
| SELE_Scal | Respiratory support or death | Inverse variance weighted (multiplicative random effects) | 6 | -0.242 | 0.031 | 0.000 |
| SELE_Scal | Respiratory support or death | Inverse variance weighted (fixed effects) | 6 | -0.242 | 0.047 | 0.000 |
| SELE_Scal | Respiratory support or death | Simple mode | 6 | -0.159 | 0.116 | 0.227 |
| SELE_Scal | Respiratory support or death | Weighted mode | 6 | -0.221 | 0.052 | 0.008 |
|  |  |  |  |  |  |  |
| sICAM1_Sliz | Respiratory support or death | Maximum likelihood | 31 | -0.174 | 0.042 | 0.000 |
| sICAM1_Sliz | Respiratory support or death | MR Egger | 31 | -0.191 | 0.082 | 0.026 |
| sICAM1_Sliz | Respiratory support or death | Simple median | 31 | -0.157 | 0.073 | 0.031 |
| sICAM1_Sliz | Respiratory support or death | Weighted median | 31 | -0.171 | 0.067 | 0.011 |
| sICAM1_Sliz | Respiratory support or death | Inverse variance weighted | 31 | -0.172 | 0.046 | 0.000 |
| sICAM1_Sliz | Respiratory support or death | IVW radial | 31 | -0.172 | 0.046 | 0.000 |
| sICAM1_Sliz | Respiratory support or death | Inverse variance weighted (multiplicative random effects) | 31 | -0.172 | 0.046 | 0.000 |
| sICAM1_Sliz | Respiratory support or death | Inverse variance weighted (fixed effects) | 31 | -0.172 | 0.041 | 0.000 |
| sICAM1_Sliz | Respiratory support or death | Simple mode | 31 | -0.102 | 0.118 | 0.397 |
| sICAM1_Sliz | Respiratory support or death | Weighted mode | 31 | -0.207 | 0.080 | 0.015 |
|  |  |  |  |  |  |  |
| SELE_Folk | Respiratory support or death | Maximum likelihood | 16 | -0.188 | 0.031 | 0.000 |
| SELE_Folk | Respiratory support or death | MR Egger | 16 | -0.107 | 0.058 | 0.086 |
| SELE_Folk | Respiratory support or death | Simple median | 16 | -0.224 | 0.058 | 0.000 |
| SELE_Folk | Respiratory support or death | Weighted median | 16 | -0.181 | 0.038 | 0.000 |
| SELE_Folk | Respiratory support or death | Inverse variance weighted | 16 | -0.186 | 0.030 | 0.000 |
| SELE_Folk | Respiratory support or death | IVW radial | 16 | -0.187 | 0.025 | 0.000 |
| SELE_Folk | Respiratory support or death | Inverse variance weighted (multiplicative random effects) | 16 | -0.186 | 0.025 | 0.000 |
| SELE_Folk | Respiratory support or death | Inverse variance weighted (fixed effects) | 16 | -0.186 | 0.030 | 0.000 |
| SELE_Folk | Respiratory support or death | Simple mode | 16 | -0.208 | 0.075 | 0.014 |
| SELE_Folk | Respiratory support or death | Weighted mode | 16 | -0.184 | 0.037 | 0.000 |
|  |  |  |  |  |  |  |
| SELE_Breth | Respiratory support or death | Maximum likelihood | 6 | -0.207 | 0.049 | 0.000 |
| SELE_Breth | Respiratory support or death | MR Egger | 6 | -0.263 | 0.136 | 0.126 |
| SELE_Breth | Respiratory support or death | Simple median | 6 | -0.129 | 0.072 | 0.071 |
| SELE_Breth | Respiratory support or death | Weighted median | 6 | -0.206 | 0.056 | 0.000 |
| SELE_Breth | Respiratory support or death | Inverse variance weighted | 6 | -0.199 | 0.066 | 0.003 |
| SELE_Breth | Respiratory support or death | IVW radial | 6 | -0.201 | 0.066 | 0.002 |
| SELE_Breth | Respiratory support or death | Inverse variance weighted (multiplicative random effects) | 6 | -0.199 | 0.066 | 0.003 |
| SELE_Breth | Respiratory support or death | Inverse variance weighted (fixed effects) | 6 | -0.199 | 0.046 | 0.000 |
| SELE_Breth | Respiratory support or death | Simple mode | 6 | -0.002 | 0.128 | 0.986 |
| SELE_Breth | Respiratory support or death | Weighted mode | 6 | -0.236 | 0.059 | 0.011 |
|  |  |  |  |  |  |  |
| PECAM1_Folk | Respiratory support or death | Maximum likelihood | 8 | -0.269 | 0.056 | 0.000 |
| PECAM1_Folk | Respiratory support or death | MR Egger | 8 | -0.171 | 0.143 | 0.278 |
| PECAM1_Folk | Respiratory support or death | Simple median | 8 | -0.236 | 0.083 | 0.004 |
| PECAM1_Folk | Respiratory support or death | Weighted median | 8 | -0.349 | 0.074 | 0.000 |
| PECAM1_Folk | Respiratory support or death | Inverse variance weighted | 8 | -0.263 | 0.055 | 0.000 |
| PECAM1_Folk | Respiratory support or death | IVW radial | 8 | -0.265 | 0.055 | 0.000 |
| PECAM1_Folk | Respiratory support or death | Inverse variance weighted (multiplicative random effects) | 8 | -0.263 | 0.055 | 0.000 |
| PECAM1_Folk | Respiratory support or death | Inverse variance weighted (fixed effects) | 8 | -0.263 | 0.054 | 0.000 |
| PECAM1_Folk | Respiratory support or death | Simple mode | 8 | -0.108 | 0.130 | 0.434 |
| PECAM1_Folk | Respiratory support or death | Weighted mode | 8 | -0.342 | 0.087 | 0.006 |
|  |  |  |  |  |  |  |
| PECAM1_Scal | Respiratory support or death | Maximum likelihood | 31 | -0.312 | 0.052 | 0.000 |
| PECAM1_Scal | Respiratory support or death | MR Egger | 31 | -0.315 | 0.087 | 0.001 |
| PECAM1_Scal | Respiratory support or death | Simple median | 31 | -0.322 | 0.106 | 0.002 |
| PECAM1_Scal | Respiratory support or death | Weighted median | 31 | -0.318 | 0.067 | 0.000 |
| PECAM1_Scal | Respiratory support or death | Inverse variance weighted | 31 | -0.314 | 0.056 | 0.000 |
| PECAM1_Scal | Respiratory support or death | IVW radial | 31 | -0.314 | 0.056 | 0.000 |
| PECAM1_Scal | Respiratory support or death | Inverse variance weighted (multiplicative random effects) | 31 | -0.314 | 0.056 | 0.000 |
| PECAM1_Scal | Respiratory support or death | Inverse variance weighted (fixed effects) | 31 | -0.314 | 0.052 | 0.000 |
| PECAM1_Scal | Respiratory support or death | Simple mode | 31 | -0.269 | 0.168 | 0.121 |
| PECAM1_Scal | Respiratory support or death | Weighted mode | 31 | -0.316 | 0.064 | 0.000 |

Number of SNPS = SNPs / Beta = BETA / Standard Error = SE / P-value = P

*ABO = ABO system transferase; C1GALT1C1 = C1GALT1 specific chaperone 1; CD207 = langerin; GCNT4 = glucosaminyl (N-Acetyl) transferase 4; LCTL = Lactase-like protein; PECAM1 = platelet endothelial cell adhesion molecule; RAB14 = ras-related protein rab-14; SELE = E-selectin; SELL =  L-selectin; sICAM1= Soluble intercellular adhesion molecule-1.*
