# Supplementary material for: Proteome-wide Mendelian randomization identifies causal links between blood proteins and severe COVID-19
Source: PLoS Genet. 2022 Mar 3;18(3):e1010042. doi: 10.1371/journal.pgen.1010042 (PMC8893330; doi:10.1371/journal.pgen.1010042)
Supplement: S6 Table — (DOCX) [file pgen.1010042.s006.docx]

# S6 Table. Table indicating the heterogeneous SNP used as instruments for at least 2 biomarkers identified in the hospitalization from COVID-19 GWAS

| **rsid** | **blood_marker** | **chromosome** |
| --- | --- | --- |
| rs11244015 | SELE_Scal, PECAM1_Scal | 9 |
| rs11523306 | C1GALT1C1_Sun, SELE_Folk, PECAM1_Scal | 9 |
| rs115478735 | FAM96A_Sun, RAB14_Sun, C1GALT1C1_Sun, SELL_Sun, KEL_Sun | 9 |
| rs117322380 | CD207_Sun, ABO_Sun | 9 |
| rs183853102 | CD207_Sun, ABO_Sun | 9 |
| rs28463601 | RAB14_Sun, SELE_Folk, KEL_Sun, SELE_Scal, PECAM1_Scal | 9 |
| rs3124762 | RAB14_Sun, ABO_Sun | 9 |
| rs3124767 | SELE_Folk, PECAM1_Scal | 9 |
| rs35434910 | GCNT4_Sun, CD207_Sun | 9 |
| rs41297217 | RAB14_Sun, ABO_Sun, SELE_Scal | 9 |
| rs4348570 | C1GALT1C1_Sun, SELL_Sun, SELE_Folk, KEL_Sun, SELE_Scal, SELE_Breth | 9 |
| rs495828 | SELE_Folk, PECAM1_Scal | 9 |
| rs4962096 | CD207_Sun, SELE_Folk | 9 |
| rs532861 | GCNT4_Sun, ABO_Sun | 9 |
| rs55988407 | CD207_Sun, RAB14_Sun, SELL_Sun | 9 |
| rs600038 | SELE_Scal, SELE_Breth | 9 |
| rs61696148 | SELL_Sun, KEL_Sun | 9 |
| rs62576042 | ABO_Sun, SELE_Scal, PECAM1_Scal | 9 |
| rs652600 | RAB14_Sun, SELL_Sun | 9 |
| rs671537 | GCNT4_Sun, ABO_Sun | 9 |
| rs68032997 | CD207_Sun, ABO_Sun, SELE_Folk, SELE_Scal | 9 |
| rs7027827 | FAM96A_Sun, RAB14_Sun | 9 |
| rs71503180 | CD207_Sun, ABO_Sun | 9 |
| rs72779222 | ABO_Sun, SELE_Scal | 9 |
| rs7869258 | FAM96A_Sun, RAB14_Sun | 9 |
| rs79158370 | FAM96A_Sun, RAB14_Sun, C1GALT1C1_Sun, SELL_Sun, SELE_Folk, KEL_Sun, SELE_Scal, PECAM1_Scal | 9 |
| rs79918022 | CD207_Sun, ABO_Sun | 9 |
| rs8176707 | SELL_Sun, SELE_Folk, SELE_Scal | 9 |
| rs8176719 | FAAH2_Sun, GCNT4_Sun, CD207_Sun, ABO_Sun | 9 |
| rs11220490 | SELE_Scal, PECAM1_Scal | 11 |
| rs11600151 | SELE_Scal, PECAM1_Scal | 11 |
| rs11671705 | GCNT4_Sun, FAM96A_Sun | 19 |
| rs601338 | GCNT4_Sun, FAM96A_Sun | 19 |
| rs62143197 | GCNT4_Sun, C1GALT1C1_Sun, ATP2A3_Sun | 19 |

This table displays the SNPs used as instruments in two or more blood proteins associated with higher/lower odds of COVID-19 hospitalization. ABO = ABO system transferase; ATP2A3 = ATPase Sarcoplasmic/Endoplasmic Reticulum Ca2+ Transporting 3; C1GALT1C1 = C1GALT1 specific chaperone 1; CD207 = langerin; FAAH2 = Fatty Acid Amide Hydrolase 2; GCNT4 = glucosaminyl (N-Acetyl) transferase 4; KEL = Kell Metallo-Endopeptidase (Kell Blood Group); LCTL = Lactase-like protein; PECAM1 = platelet endothelial cell adhesion molecule; RAB14 = ras-related protein rab-14; SELE = E-selectin; SELL = L-selectin; SFTPD = Surfactant Protein D.
