# Supplementary material for: Proteome-wide Mendelian randomization identifies causal links between blood proteins and severe COVID-19
Source: PLoS Genet. 2022 Mar 3;18(3):e1010042. doi: 10.1371/journal.pgen.1010042 (PMC8893330; doi:10.1371/journal.pgen.1010042)
Supplement: S7 Table — (DOCX) [file pgen.1010042.s007.docx]

## S7 Table. Table indicating the heterogeneous SNP used as instruments for at least 2 biomarkers identified in the respiratory support/death as a result of COVID-19 GWAS

| **rsid** | **blood_marker** | **chromosome** |
| --- | --- | --- |
| rs11244015 | SELE_Sliz, SELE_Scal, PECAM1_Scal | 9 |
| rs11523306 | SELE_Folk, PECAM1_Scal | 9 |
| rs11531875 | PECAM1_Folk, PECAM1_Scal | 9 |
| rs115478735 | RAB14_Sun, C1GALT1C1_Sun, SELL_Sun | 9 |
| rs117322380 | CD207_Sun, ABO_Sun | 9 |
| rs118167234 | SELE_Sliz, sICAM1_Sliz | 9 |
| rs183853102 | FAM96A_Sun, CD207_Sun, ABO_Sun | 9 |
| rs1858879 | FAM96A_Sun, C1GALT1C1_Sun | 9 |
| rs28463601 | RAB14_Sun, SELE_Scal, SELE_Folk, PECAM1_Scal | 9 |
| rs3124767 | SELE_Sliz, SELE_Folk, PECAM1_Folk, PECAM1_Scal | 9 |
| rs34512234 | GCNT4_Sun, FAM96A_Sun, ABO_Sun | 9 |
| rs41297217 | RAB14_Sun, ABO_Sun, SELE_Scal | 9 |
| rs4348570 | C1GALT1C1_Sun, SELL_Sun, SELE_Scal, SELE_Breth | 9 |
| rs4489379 | FAM96A_Sun, ABO_Sun | 9 |
| rs495828 | SELE_Folk, PECAM1_Folk, PECAM1_Scal | 9 |
| rs4962096 | CD207_Sun, SELE_Folk | 9 |
| rs4962127 | RAB14_Sun, SELE_Sliz | 9 |
| rs57184987 | ABO_Sun, SELE_Scal | 9 |
| rs576123 | GCNT4_Sun, FAM96A_Sun, CD207_Sun, ABO_Sun | 9 |
| rs600038 | SELE_Sliz, SELE_Scal, SELE_Breth | 9 |
| rs62575992 | RAB14_Sun, CD207_Sun, SELE_Sliz | 9 |
| rs62576042 | SELE_Scal, PECAM1_Scal | 9 |
| rs652600 | RAB14_Sun, SELL_Sun | 9 |
| rs68032997 | CD207_Sun, SELE_Scal, SELE_Folk | 9 |
| rs7852396 | CD207_Sun, SELE_Folk | 9 |
| rs79158370 | RAB14_Sun, C1GALT1C1_Sun, SELE_Sliz, SELL_Sun, SELE_Scal, SELE_Folk, PECAM1_Folk, PECAM1_Scal | 9 |
| rs79918022 | CD207_Sun, ABO_Sun | 9 |
| rs8176686 | RAB14_Sun, C1GALT1C1_Sun, SELE_Sliz, sICAM1_Sliz, SELE_Folk | 9 |
| rs8176707 | SELL_Sun, SELE_Scal | 9 |
| rs8176720 | GCNT4_Sun, FAM96A_Sun, ABO_Sun | 9 |
| rs11220490 | SELE_Scal, PECAM1_Scal | 11 |
| rs11600151 | SELE_Scal, PECAM1_Scal | 11 |
| rs11671705 | GCNT4_Sun, FAM96A_Sun | 19 |
| rs601338 | GCNT4_Sun, FAM96A_Sun | 19 |
| rs62143197 | GCNT4_Sun, C1GALT1C1_Sun | 19 |

This table displays the SNPs used as instruments in two or more blood proteins associated with higher/lower odds of respiratory support/death as a result of COVID-19. ABO = ABO system transferase; C1GALT1C1 = C1GALT1 specific chaperone 1; CD207 = langerin; GCNT4 = glucosaminyl (N-Acetyl) transferase 4; LCTL = Lactase-like protein; PECAM1 = platelet endothelial cell adhesion molecule; RAB14 = ras-related protein rab-14; SELE = E-selectin; SELL = L-selectin; sICAM1= Soluble intercellular adhesion molecule-1.
