# Supplementary material for: Proteome-wide Mendelian randomization identifies causal links between blood proteins and severe COVID-19
Source: PLoS Genet. 2022 Mar 3;18(3):e1010042. doi: 10.1371/journal.pgen.1010042 (PMC8893330; doi:10.1371/journal.pgen.1010042)
Supplement: S8 Table — (DOCX) [file pgen.1010042.s008.docx]

# S8 Table. cis-SNP effects from the significantly associated proteins

| **Outcome - Hospitalization as a result of COVID-19** | | | | |  |
| --- | --- | --- | --- | --- | --- |
|  |  |  |  |  |  |
| **Protein (exposure)** | **BETA** | **SE** | **P** | **SNPs** |  |
| ABO_Sun | 0.08 | 0.01 | 0.00 | 17 |  |
| SFTPD_Breth | -0.11 | 0.03 | 0.00 | 6 |  |
| SELE_Scal | 0.09 | 0.30 | 0.77 | 2 |  |
|  |  |  |  |  |  |
|  |  |  |  |  |  |
| **Outcome - Respiratory support/death as a result of COVID-19** | | | | |  |
|  |  |  |  |  |  |
| **Protein (exposure)** | **BETA** | **SE** | **P** | **SNPs** |  |
| ABO_Sun | 0.13 | 0.02 | 0.00 | 18 |  |
| SELE_Scal | 0.09 | 0.30 | 0.77 | 2 |  |
| SELE_Sliz | 0.21 | 0.33 | 0.52 | 1 |  |
| sICAM1_Sliz | -0.14 | 0.06 | 0.03 | 18 |  |

Number of SNPS = SNPs / Beta = BETA / Standard Error = SE / P-value = P

This table displays associations between blood proteins that we find to have a significant effect (exposure) and COVID-19 phenotypes (outcome), using only cis-SNPs as genetic instruments. We did not identify cis-SNPs for other blood proteins.
