# Supplementary material for: Proteome-wide Mendelian randomization identifies causal links between blood proteins and severe COVID-19
Source: PLoS Genet. 2022 Mar 3;18(3):e1010042. doi: 10.1371/journal.pgen.1010042 (PMC8893330; doi:10.1371/journal.pgen.1010042)
Supplement: S9 Table — (DOCX) [file pgen.1010042.s009.docx]

# S9 Table. COVID-19 associations with BMI

| **Exposure** | **Outcome** | **Beta** | **SE** | **p value** | **SNPs** | **OR** | **Lower 95% CI** | **Higher 95% CI** |
| --- | --- | --- | --- | --- | --- | --- | --- | --- |
| BMI | Hospitalized_GWAS | 0.365 | 0.044 | 5.45E-17 | 1183 | 1.441 | 1.323 | 1.570 |
| BMI | Severe_GWAS | 0.316 | 0.066 | 1.59E-06 | 1177 | 1.372 | 1.206 | 1.561 |
| Hospitalized_GWAS | BMI | 0.016 | 0.004 | 1.79E-05 | 18 | 1.016 | 1.009 | 1.023 |
| Severe_GWAS | BMI | 0.004 | 0.002 | 7.05E-02 | 18 | 1.004 | 1.000 | 1.009 |

Number of SNPS = SNPs / Beta = BETA / Standard Error = SE / P-value = P

This table displays bi-directional associations between a genetic propensity for higher BMI and COVID-19 (Hospitalized and Severe). When BMI is the exposure, the p-value for identifying SNP instruments was set to p < 10x5E8; when COVID-19 is the exposure, the p-value for identifying SNP instruments was set to p < 5x10e6.

# 
