# Supplementary material for: Proteome-wide Mendelian randomization identifies causal links between blood proteins and severe COVID-19
Source: PLoS Genet. 2022 Mar 3;18(3):e1010042. doi: 10.1371/journal.pgen.1010042 (PMC8893330; doi:10.1371/journal.pgen.1010042)
Supplement: S10 Table — (DOCX) [file pgen.1010042.s010.docx]

# S10 Table. Blood marker associations with BMI

| **Exposure - Body Mass Index** | | | | | | | | |  |
| --- | --- | --- | --- | --- | --- | --- | --- | --- | --- |
|  |  |  |  |  |  |  |  |  |  |
| **Protein (outcome)** | **Beta** | **SE** | **p value** | **SNPs** | **OR** | **Lower 95% CI** | **Upper 95% CI** | **q value** |  |
| SELE_Scal | 0.144 | 0.021 | 0.000 | 1212 | 1.155 | 1.114 | 1.197 | 0.000 |  |
| C1GALT1C1_Sun | -0.198 | 0.056 | 0.000 | 1213 | 0.821 | 0.712 | 0.930 | 0.003 |  |
| SELE_Folk | 0.223 | 0.063 | 0.000 | 1070 | 1.250 | 1.125 | 1.374 | 0.003 |  |
| KEL_Sun | 0.170 | 0.056 | 0.002 | 1208 | 1.185 | 1.075 | 1.294 | 0.011 |  |
| SELL_Sun | 0.147 | 0.056 | 0.008 | 1207 | 1.158 | 1.049 | 1.268 | 0.030 |  |
| RAB14_Sun | -0.132 | 0.056 | 0.018 | 1210 | 0.876 | 0.767 | 0.985 | 0.048 |  |
| SFTPD_Breth | -0.195 | 0.083 | 0.019 | 1196 | 0.822 | 0.659 | 0.986 | 0.048 |  |
| ABO_Sun | -0.105 | 0.056 | 0.059 | 1210 | 0.900 | 0.791 | 1.009 | 0.132 |  |
| SELE_Breth | 0.139 | 0.076 | 0.069 | 1194 | 1.149 | 0.999 | 1.298 | 0.139 |  |
| GCNT4_Sun | 0.097 | 0.056 | 0.081 | 1213 | 1.102 | 0.993 | 1.211 | 0.146 |  |
| FAAH2_Sun | -0.087 | 0.056 | 0.119 | 1207 | 0.917 | 0.807 | 1.026 | 0.194 |  |
| CD207_Sun | -0.073 | 0.056 | 0.190 | 1206 | 0.929 | 0.820 | 1.039 | 0.286 |  |
| PECAM1_Scal | 0.027 | 0.021 | 0.214 | 1210 | 1.027 | 0.985 | 1.069 | 0.296 |  |
| ATP2A3_Sun | -0.060 | 0.056 | 0.284 | 1210 | 0.942 | 0.833 | 1.051 | 0.365 |  |
| PECAM1_Folk | 0.063 | 0.063 | 0.323 | 1075 | 1.065 | 0.941 | 1.189 | 0.387 |  |
| sICAM1_Sliz | 0.040 | 0.045 | 0.367 | 1209 | 1.041 | 0.953 | 1.129 | 0.413 |  |
| SELE_Sliz | -0.020 | 0.045 | 0.651 | 1213 | 0.980 | 0.892 | 1.068 | 0.690 |  |
| LCTL_Sun | 0.021 | 0.056 | 0.707 | 1214 | 1.021 | 0.912 | 1.130 | 0.707 |  |
|  |  |  |  |  |  |  |  |  |  |
| **Outcome - Body Mass Index** | | | | | | | | |  |
|  |  |  |  |  |  |  |  |  |  |
| **Protein (exposure)** | **Beta** | **SE** | **p value** | **SNPs** | **OR** | **Lower 95% CI** | **Upper 95% CI** | **q value** |  |
| LCTL_Sun | 0.013 | 0.002 | 0.000 | 25 | 1.013 | 1.010 | 1.016 | 0.000 |  |
| SELE_Sliz | -0.005 | 0.002 | 0.002 | 42 | 0.995 | 0.992 | 0.998 | 0.017 |  |
| SFTPD_Breth | 0.007 | 0.002 | 0.006 | 7 | 1.007 | 1.002 | 1.011 | 0.035 |  |
| PECAM1_Scal | -0.009 | 0.003 | 0.009 | 18 | 0.991 | 0.984 | 0.998 | 0.036 |  |
| RAB14_Sun | 0.005 | 0.002 | 0.014 | 10 | 1.005 | 1.001 | 1.009 | 0.045 |  |
| SELE_Scal | -0.005 | 0.002 | 0.031 | 31 | 0.995 | 0.991 | 1.000 | 0.082 |  |
| ATP2A3_Sun | -0.011 | 0.006 | 0.071 | 6 | 0.989 | 0.976 | 1.001 | 0.147 |  |
| SELE_Folk | -0.004 | 0.002 | 0.073 | 8 | 0.996 | 0.992 | 1.000 | 0.147 |  |
| KEL_Sun | -0.005 | 0.003 | 0.096 | 9 | 0.995 | 0.990 | 1.001 | 0.162 |  |
| GCNT4_Sun | 0.004 | 0.003 | 0.101 | 10 | 1.004 | 0.999 | 1.009 | 0.162 |  |
| C1GALT1C1_Sun | 0.004 | 0.002 | 0.115 | 16 | 1.004 | 0.999 | 1.009 | 0.167 |  |
| ABO_Sun | 0.002 | 0.001 | 0.199 | 17 | 1.002 | 0.999 | 1.004 | 0.265 |  |
| CD207_Sun | 0.002 | 0.002 | 0.225 | 12 | 1.002 | 0.999 | 1.006 | 0.277 |  |
| SELL_Sun | -0.002 | 0.002 | 0.266 | 13 | 0.998 | 0.994 | 1.002 | 0.304 |  |
| sICAM1_Sliz | -0.003 | 0.003 | 0.410 | 13 | 0.997 | 0.990 | 1.004 | 0.437 |  |
| FAAH2_Sun | -0.001 | 0.003 | 0.735 | 6 | 0.999 | 0.993 | 1.005 | 0.735 |  |

Number of SNPS = SNPs / Beta = BETA / Standard Error = SE / P-value = P

The top table displays associations between a genetic propensity for higher BMI (exposure) and blood marker levels (outcome). The bottom table displays associations between a genetic propensity for higher blood marker levels (exposure) and BMI (outcome). Significant associations are highlighted in green (Q < 0.05).
