# Supplementary material for: Proteome-wide Mendelian randomization identifies causal links between blood proteins and severe COVID-19
Source: PLoS Genet. 2022 Mar 3;18(3):e1010042. doi: 10.1371/journal.pgen.1010042 (PMC8893330; doi:10.1371/journal.pgen.1010042)
Supplement: S11 Table — (DOCX) [file pgen.1010042.s011.docx]

# S11 Table. Details on the tissue, function, and Covid-19 relevance of each significant blood biomarker

| **Name** | **Gene** | **Tissue** | **Function** | **Covid-19 hypothesis** |
| --- | --- | --- | --- | --- |
| ABO system transferase | ABO | Blood | This gene encodes proteins related to the first discovered blood group system, ABO.   Variation in the ABO gene is the basis of the ABO blood group, thus the presence of an allele determines the blood group in an individual.   The 'O' blood group is caused by a deletion of guanine-258 near the N-terminus of the protein which results in a frameshift and translation of an almost entirely different protein.   Individuals with the A, B, and AB alleles express glycosyltransferase activities that convert the H antigen into the A or B antigen.   This protein is the basis of the ABO blood group system. The histo-blood group ABO involves three carbohydrate antigens: A, B, and H. A, B, and AB individuals express a glycosyltransferase activity that converts the H antigen to the A antigen (by addition of UDP-GalNAc) or to the B antigen (by addition of UDP-Gal), whereas O individuals lack such activity. | This locus has been identified as a susceptibility locus for severe coronavirus disease 2019 (COVID-19) by genome-wide association study.   Patients with blood group A had an increased risk for infection with SARS-CoV-2, whereas blood group O was associated with a decreased risk, indicating that certain ABO blood groups were correlated with SARS-CoV-2 susceptibility [1]  Although ABO blood type and/or cardiovascular diseases are prognostic of COVID-19 patient severity, they are not risk factors predisposing to the risk of getting SARS-CoV-2 infection [2]  No association between ABO type and death among individuals hospitalized with COVID-19 (X2 = 1.35, p=0.717) [3]   Associations between ABO blood groups and COVID-19 susceptibility. The COVID-19 risk significantly increased for blood group A (OR 1.279, 95% CI 1.136~1.440) and decreased for blood group O (OR 0.680, 95% CI .599~.771) [4]  Blood type A might be more susceptible to infect COVID-19 while blood type O might be less susceptible to infect COVID-19 [5]  Critically ill COVID-19 patients with blood group A or AB are at increased risk for requiring mechanical ventilation, CRRT, and prolonged ICU admission compared with patients with blood g[roup O or B](https://ashpublications.org/bloodadvances/article/4/20/4981/464437) [6] |
| Sarcoplasmic/endoplasmic reticulum calcium ATPase 3 | ATP2A3 | Found in most tissues. Most abundant in thymus, trachea, salivary gland, spleen, bone marrow, lymph node, peripheral leukocytes, pancreas and colon. | This magnesium-dependent enzyme catalyzes the hydrolysis of ATP coupled with the transport of calcium.  Transports calcium ions from the cytosol into the sarcoplasmic/endoplasmic reticulum lumen.  Contributes to calcium sequestration involved in muscular excitation/contraction. | Physical Frailty/Sarcopenia as a Key Predisposing Factor to Coronavirus Disease 2019 (COVID-19) and Its Complications in Older Adults [7] |
| C1GALT1 specific chaperone 1 | C1GALT1C1 | Ubiquitously expressed. Abundantly expressed in salivary gland, stomach, small intestine, kidney, and testis and at intermediate levels in whole brain, cerebellum, spinal cord, thymus, spleen, trachea, lung, pancreas, ovary, and uterus | This gene encodes a type II transmembrane protein that is similar to the core 1 beta1,3-galactosyltransferase 1, which catalyzes the synthesis of the core-1 structure, also known as Thomsen-Friedenreich antigen, on O-linked glycans.   This gene product lacks the galactosyltransferase activity itself, but instead acts as a molecular chaperone required for the folding, stability and full activity of the core 1 beta1,3-galactosyltransferase 1.   Mutations in this gene have been associated with Tn syndrome  Involved in protein binding and glycoprotein-N-acetylgalactosamine 3-beta-galactosyltransferase activity | A positive association between predicted protein level and COVID-19 severity was detected for C1GALT1C1 [8] |
| Langerin | CD207 | Exclusively expressed by Langerhans cells. Expressed in astrocytoma and malignant ependymoma, but not in normal brain tissues. | The protein encoded by this gene is expressed only in Langerhans cells which are immature dendritic cells of the epidermis and mucosa.   It is localized in the Birbeck granules, organelles present in the cytoplasm of Langerhans cells and consisting of superimposed and zippered membranes.   It is a C-type lectin with mannose binding specificity, and it has been proposed that mannose binding by this protein leads to internalization of antigen into Birbeck granules and providing access to a nonclassical antigen-processing pathway.   Induces the formation of Birbeck granules (BGs); is a potent regulator of membrane superimposition and zippering. Binds to sulfated as well as mannosylated glycans, keratan sulfate (KS) and beta-glucans.   Facilitates uptake of antigens and is involved in the routing and/or processing of antigen for presentation to T cells.  Protects against human immunodeficiency virus-1 (HIV-1) infection.   Binds to high-mannose structures present on the envelope glycoprotein which is followed by subsequent targeting of the virus to the Birbeck granules leading to its rapid degradation. |  |
| Fatty-acid amide hydrolase 2 | FAAH2 | Expressed in kidney, liver, lung, prostate, heart, and ovary | Catalyses the hydrolysis of endogenous amidated lipids like the sleep-inducing lipid oleamide ((9Z)-octadecenamide), the endocannabinoid anandamide (N-(5Z,8Z,11Z,14Z-eicosatetraenoyl)-ethanolamine), as well as other fatty amides, to their corresponding fatty acids, thereby regulating the signalling functions of these molecules.  Degrades [endocannabinoids](https://en.wikipedia.org/wiki/Endocannabinoid) and defects in this enzyme have been associated with neurologic and psychiatric disorders  Hydrolyses monounsaturated substrate anandamide preferentially as compared to polyunsaturated substrates. | Coronavirus-induced severe acute respiratory syndrome as a possible expression of fatty acid amide hydrolase hyperactivation and possible therapeutic role of FAAH inhibitors in COVID-19 [9]  The Endocannabinoid System as Prognostic Biomarker of the Obstructive Sleep Apnea Morbidity in COVID-19-Recovered Individuals [10]  Viral block of ACE2 activity could activate the fatty acid amide hydrolase (FAAH), the enzyme involved in the destruction of cannabinoids, with a following endocannabinoid system deficiency. A Preliminary Phase 2 Study with Angiotensin 1-7 in Association with Melatonin and Cannabidiol in Symptomatic COVID19 -Infected Subjects [11]  In fact, studies using FAAH inhibitor have shown that such approaches lead to an increase in AEA and consequent suppression of autoimmune hepatitis triggered by a polyclonal activator of T cells which triggers cytokine storm: Use of Cannabinoids to Treat Acute Respiratory Distress Syndrome and Cytokine Storm Associated with Coronavirus Disease-2019 [12] |
| Glucosaminyl (N-Acetyl) transferase 4 | GCNT4 | Omnipresent, hight expression in GI-tract, endocrine tissues and blood | Invovled in carbohydrate metabolic processes  Invovled in protein glycosylation and protein O-linked glycosylation   Involved in O-glycan processing  Transferase activity, transferring glycosyl groups   Acetylglucosaminyltransferase activity   N-acetyllactosaminide beta-1,6-N-acetylglucosaminyltransferase activity   beta-1,3-galactosyl-O-glycosyl-glycoprotein beta-1,6-N-acetylglucosaminyltransferase activity |  |
| Kell Metallo-Endopeptidase (Kell Blood Group) | KEL | Expressed at high levels in erythrocytes and testis (in Sertoli cells), and, at lower levels, in skeletal muscle, tonsils (in follicular dendritic cells), lymph node, spleen and appendix (at protein level). | Zinc endopeptidase with endothelin-3-converting enzyme activity.   Cleaves EDN1, EDN2 and EDN3, with a marked preference for EDN3.  The Kell antigen system is a human blood group system, that is, group of antigens on the human red blood cell surface which are important determinants of blood type and are targets for autoimmune or alloimmune diseases which destroy red blood cells. | Kell negative phenotype found to be more susceptible to COVID-19 [13] |
| Lactase-like protein | LCTL | Expressed in sural nerve and 80 other tissues | Lactase-like is a glycosidase enzyme.  Plays a role in formation of the lens suture in the eye, which is important for normal optical properties of the lens. |  |
| Macrophage inflammatory protein | MIP1b | Enriched expression in blood, lung, lymphoid tissue | Crucial for immune responses towards infection and inflammation  MIP-1 are best known for their chemotactic and proinflammatory effects but can also promote homeostasis. | Co0005 C-C Chemokine Receptor Type 5 And Its Ligands Ccl4, 8 And 11 Can Link Covid-19, Rheumatoid Arthritis And Hydroxychloroquine [14]  CCL4 were in significantly lower levels in COVID patients than healthy controls [15]  Macrophage inflammatory protein-1β (MIP1b; more commonly known as Chemokine (C-C motif) ligands 4 (CCL4) was inversely associated with COVID-19 [odds ratio (OR) 0.97 per SD, 95% confidence interval (CI) 0.96–0.99] but not after adjustment for multiple comparisons. This finding replicated for hospitalized COVID-19 (OR 0.93, 95% CI 0.89–0.98) [16] |
| Neprilysin | NEP | Blood, intestine, kidney & 150+ other tissues | Able to cleave angiotensin-1, angiotensin-2 and angiotensin 1-9  Involved in the degradation of atrial natriuretic factor and brain natriuretic factor  Important cell surface marker in the diagnostic of human acute lymphocytic leukemia | Considering the protective effect of NEP against pulmonary inflammatory reactions and fibrosis, it is suggested to direct the future efforts towards its potential role in COVID-19 pathophysiology [17]  NEP-based therapeutic properties of roflumilast, which may be of great importance in curing COVID-19 [18]  NEP/angiotensin receptor type 1 (AT1R) inhibitor sacubitril/valsartan (SAC/VAL) may increase levels of these molecules and block AT1Rs required for ACE2 endocytosis in SARS-CoV-2 infection [19]  Therefore, ACE2-Neprilysin-CA complex could be the key factor of pathogenesis of SARS-CoV-2 and may provide us useful information to find better provocative and therapeutic strategies against COVID-19 [20]  Neprilysin (NEP) and angiotensin-converting enzyme (ACE) have emerged as the pharmaceutical targets of interest in the search for therapeutic interventions against COVID-19 [21] |
| Ras-related protein rab-14 | RAB14 | Expressed in upper lobe of lung, endocrine tissues, GI tract, bone marrow and lymphoid tissue | RAB14 belongs to the large RAB family of low molecular mass GTPases that are involved in intracellular membrane trafficking   Involved in membrane trafficking between the Golgi complex and endosomes during early embryonic development.   Regulates the Golgi to endosome transport of FGFR-containing vesicles during early development, a key process for developing basement membrane and epiblast and primitive endoderm lineages during early postimplantation development.   May act by modulating the kinesin KIF16B-cargo association to endosomes (By similarity).   Regulates, together with its guanine nucleotide exchange factor DENND6A, the specific endocytic transport of ADAM10, N-cadherin/CDH2 shedding and cell-cell adhesion. |  |
| Platelet endothelial cell adhesion molecule | PECAM-1 | Expressed on platelets and leukocytes and is primarily concentrated at the borders between endothelial cells | The protein encoded by this gene is found on the surface of platelets, monocytes, neutrophils, and some types of T-cells, and makes up a large portion of endothelial cell intercellular junctions.   The encoded protein is a member of the immunoglobulin superfamily and is likely involved in leukocyte migration, angiogenesis, and integrin activation  Among its related pathways are Degradation of the extracellular matrix and Platelet homeostasis  Cell adhesion molecule which is required for leukocyte transendothelial migration (TEM) under most inflammatory conditions   Promotes macrophage-mediated phagocytosis of apoptotic leukocytes by tethering them to the phagocytic cells; PECAM1-mediated detachment signal appears to be disabled in apoptotic leukocytes | Progression of the disease, but not the virus itself, damages the endothelium, leading to the elevated levels of endothelial-specific adhesion molecules, including sPECAM-1. [22]  A low expression of adhesion molecules together with a high expression of inhibitory receptors in neutrophils from children with COVID-19 might prevent tissue infiltration by neutrophils preserving lung functio[n](https://www.sciencedirect.com/science/article/pii/S235239642100150X) [23] |
| L-selectin | SELL | Expressed in B-cell lines and T-lymphocytes | This gene encodes a cell surface adhesion molecule that belongs to a family of adhesion/homing receptors.   The encoded protein contains a C-type lectin-like domain, a calcium-binding epidermal growth factor-like domain, and two short complement-like repeats.   The gene product is required for binding and subsequent rolling of leucocytes on endothelial cells, facilitating their migration into secondary lymphoid organs and inflammation sites.   Single-nucleotide polymorphisms in this gene have been associated with various diseases including immunoglobulin A nephropathy.   Calcium-dependent lectin that mediates cell adhesion by binding to glycoproteins on neighboring cells  Mediates the adherence of lymphocytes to endothelial cells of high endothelial venules in peripheral lymph nodes. Promotes initial tethering and rolling of leukocytes in endothelia   Polymorphism in ICAM-1, PECAM-1, E-selectin, and L-selectin genes in Tunisian patients with inflammatory bowel disease | Compared with healthy controls, neutrophils from children with COVID-19 showed a lower expression of CD11b, CD66b, and L-selectin but a higher expression of the activation markers HLA-DR, CD64 and PECAM-1 and the inhibitory receptors LAIR-1 and PD-L1 [23]  The use of heparin in COVID-19 pneumonia: heparin also exhibits anti-inflammatory properties. Although still to be fully clarified, some of the proposed mechanisms include binding with inflammatory cytokines, inhibition of neutrophil chemotaxis and leukocyte migration, neutralization of complement factor C5a and sequestration of acute phase proteins such as P-selectin and L-selectin, and induction of cell apoptosis through tumor necrosis factor α and nuclear factor κB pathway[s](https://www.sciencedirect.com/science/article/pii/S2213333X20303425?casa_token=MHPBBfL4NWgAAAAA:uXhZHxSedRhUJopKni0YEBL2l__EtOLqRTwMgk-rz5Wne5WE1nsEZpKEzt76sueyyMfx6iE9APYp) [24] |
| E-selectin | SELE | Cytoplasmic expression in blood vessels and megakaryocytes in bone marrow. | The protein encoded by this gene is found in cytokine-stimulated endothelial cells and is thought to be responsible for the accumulation of blood leukocytes at sites of inflammation by mediating the adhesion of cells to the vascular lining.   It exhibits structural features such as the presence of lectin- and EGF-like domains followed by short consensus repeat (SCR) domains that contain 6 conserved cysteine residues.   These proteins are part of the selectin family of cell adhesion molecules. Adhesion molecules participate in the interaction between leukocytes and the endothelium and appear to be involved in the pathogenesis of atherosclerosis  Diseases associated with SELE include Rheumatoid Vasculitis and African Tick-Bite Fever. Among its related pathways are Cell surface interactions at the vascular wall and ATF-2 transcription factor network.   Gene Ontology (GO) annotations related to this gene include transmembrane signaling receptor activity and phospholipase binding  Cell-surface glycoprotein having a role in immunoadhesion. Mediates in the adhesion of blood neutrophils in cytokine-activated endothelium through interaction with SELPLG/PSGL1. May have a role in capillary morphogenesis | sE-selectin and angiopoietin-2 were increased in critically ill COVID-19 patients. Although angiopoietin-2 was the best biomarker to predict transfer to the ICU and was associated with poor lung compliance in COVID-19 patients [25]  In COVID-19-related ARDS, the plasma levels of Ang-2 and ICAM-1 at T1 were statistically higher in non-survivors than survivors, (p = 0.04 and p = 0.03, respectively), whereas those of P-selectin, E-selectin and RAGE did not diffe[r](https://ccforum.biomedcentral.com/articles/10.1186/s13054-021-03499-4) [26]  Significantly increased levels of VCAM-1, E-Selectin, and CD31 in COVID-19 plasma [27] |
| Surfactant protein D | SFTPD | Expressed in lung, brain, pancreas and adipose tissue | Contributes to the lung's defence against inhaled microorganisms, organic antigens, and toxins. | Two surfactant proteins (SFTPC and SFTPD) were expressed at remarkably low levels in the COVID-19 lungs [28]  3 intersection target genes (PLA2G1B, SFTPD, SLCO4C1) were related to COVID immunity [29]  SFTPD is highly expressed in the lung and predicted to interact with the spike protein of SARS-CoV-2 [30]  Expression of surfactant protein D (SFTPD), a gene encoding a protein involved in the innate immune response to protect the lungs against inhaled microorganisms and chemicals, is decreased [31] |
| Intercellular Adhesion Molecule 1 | sICAM1 | Expressed in lung and 218 other tissues | ICAM proteins are ligands for the leukocyte adhesion protein LFA-1  Acts as a receptor for major receptor group rhinovirus A-B capsid proteins  Upon Kaposi's sarcoma-associated herpesvirus/HHV-8 infection, is degraded to prevent lysis of infected cells by cytotoxic T-lymphocytes and NK cell  Cell adhesion molecules represent important biomarkers for inflammatory processes  Research has focused on their role in cardiovascular diseases | Crucial role in pathogenesis of COVID-19 related to Angiotensin -2 concentration, soluble intracellular adhesion molecules type -1 and ABO blood group [32]  Increased expression of endothelial cell adhesion molecules is related to COVID-19 disease severity and may contribute to coagulation dysfunction [33]  COVID-19 postmortem lung samples showed higher endothelial expression of ICAM-1 [34] |

C4B = complement C4B (Chido blood group), CREB3L4 = cAMP responsive element binding protein 3 like 4, HLA-DQA2 = major histocompatibility complex (MHC), class II, DQ alpha 2, MICA = MHC class I polypeptide-related sequence A, PRTN3 = proteinase 3, TNNI3 = troponin I3, cardiac type, GCNT4 = glucosaminyl (N-Acetyl) transferase 4, CAST = calpastatin, FAM96A = cytosolic iron-sulfur assembly component 2A, C1GALT1C1 = C1GALT1 specific chaperone 1, RAB14 = ras-related protein rab-14, CD207 = langerin, ABO = ABO system transferase, HMGB1 = high mobility group box protein 1, SELL = L-selectin, IL3RA = interleukin 3 receptor, alpha,  SELE = E-selectin, PECAM-1 = platelet endothelial cell adhesion molecule; C4A = complement C4A (Rodgers blood group),

**References**:

[1] Wu, Yuqin, Zhicai Feng, Peng Li, and Qizhi Yu. 2020. “Relationship between ABO Blood Group Distribution and Clinical Characteristics in Patients with COVID-19.” Clinica Chimica Acta; International Journal of Clinical Chemistry 509 (October): 220–23.

[2] Dai, Xiaofeng. 2020. “ABO Blood Group Predisposes to COVID-19 Severity and Cardiovascular Diseases.” European Journal of Preventive Cardiology 27 (13): 1436–37.

[3] Dzik, Sunny, Kent Eliason, Edward B. Morris, Richard M. Kaufman, and Crystal M. North. 2020. “COVID-19 and ABO Blood Groups.” Transfusion. Wiley.

[4] Zhao, Jiao, Yan Yang, Hanping Huang, Dong Li, Dongfeng Gu, Xiangfeng Lu, Zheng Zhang, et al. 2020. “Relationship between the ABO Blood Group and the COVID-19 Susceptibility.” Clinical Infectious Diseases: An Official Publication of the Infectious Diseases Society of America, August. https://doi.org/10.1093/cid/ciaa1150.

[5] Wu, Bing-Bing, Dong-Zhou Gu, Jia-Ning Yu, Jie Yang, and Wang-Qin Shen. 2020. “Association between ABO Blood Groups and COVID-19 Infection, Severity and Demise: A Systematic Review and Meta-Analysis.” Infection, Genetics and Evolution: Journal of Molecular Epidemiology and Evolutionary Genetics in Infectious Diseases 84 (October): 104485.

[6] Hoiland, Ryan L., Fergusson, Nicholas A., Mitra, Anish R., Griesdale, Donald E. G., Devine, Dana V., Stukas, Sophie, Cooper Jennifer, Thiara, Sonny, Foster, Denise, Chen, Luke Y. C., Lee, Agnes Y. Y., Conway, Edward M., Wellington, Cheryl L., Sekhon, Mypinder S. “The association of ABO blood group with indices of disease severity and multiorgan dysfunction in COVID-19” Blood Advances, (2020) 4 (20): 4981–4989.

[7] Ali, Amira Mohammed, and Hiroshi Kunugi. 2021. “Physical Frailty/Sarcopenia as a Key Predisposing Factor to Coronavirus Disease 2019 (COVID-19) and Its Complications in Older Adults.” BioMed 1 (1): 11–40.

[8] Zhu, Jingjing, Chong Wu, and Lang Wu. 2021. “Associations between Genetically Predicted Protein Levels and COVID-19 Severity.” The Journal of Infectious Diseases 223 (1): 19–22.

[9] Lissoni, Paolo, Franco Rovelli, Francesco Pelizzoni, Arianna Lissoni, and Giuseppe Di Fede. 2020. “Coronavirus-Induced Severe Acute Respiratory Syndrome (SARS) as a Possible Expression of Fatty Acid Amide Hydrolase (FAAH) Hyper-Function and Possible Therapeutic Role of FAAH Inhibitors in Covid 19-Induced SARS.” Primary Sensory Neuron: The International Interdisciplinary Journal Reporting Basic and Clinical Research on Sensory Receptors and Primary Afferent Neurons 5: 2690–1919.

[10] Murillo-Rodríguez, Eric. 2021. “The Endocannabinoid System as Prognostic Biomarker of the Obstructive Sleep Apnea Morbidity in COVID-19-Recovered Individuals.” Sleep and Vigilance, September, 1–7.

[11] Lissoni, Paolo, Franco Rovelli, Alejandra Monzon, Giusy Messina, Enrica Porta, Giorgio Porro, Sonia Pensato, et al. 2020. “COVID-19 Disease as an Acute Angiotensin 1-7 Deficiency: A Preliminary Phase 2 Study with Angiotensin 1-7 in Association with Melatonin and Cannabidiol in Symptomatic COVID19 -Infected Subjects.” Journal of Infectiology 3 (2). https://www.infectiologyjournal.com/articles/covid-19-disease-as-an-acute-angiotensin-1-7-deficiency-a-preliminary-phase-2-study-with-angiotensin-1-7-in-association-with-melatonin-and-cannabidiol-in-symptomatic-covid19-infected-subjects.

[12] Nagarkatti, Prakash, Kathryn Miranda, and Mitzi Nagarkatti. 2020. “Use of Cannabinoids to Treat Acute Respiratory Distress Syndrome and Cytokine Storm Associated with Coronavirus Disease-2019.” Frontiers in Pharmacology 11 (November): 589438.

[13] Bhandari, Sudhir, Ajit Shaktawat Singh, Amit Tak, Jyotsna Shukla, Jitentdra Gupta, Bhoopendra Patel, Shivankan Kakkar, et al. 2020. “Relationship between ABO Blood Group Phenotypes and NCOVID-19 Susceptibility: A Retrospective Observational Study.” Scripta Medica 51 (4): 217–39.

[14] Hachim, M. Y., and S. Hannawi. 2020. “Co0005 C-c Chemokine Receptor Type 5 and Its Ligands Ccl4, 8 and 11 Can Link Covid-19, Rheumatoid Arthritis and Hydroxychloroquine.” Annals of the Rheumatic Diseases 79 (Suppl 1): 213.3-214.

[15] Patterson, Bruce K., Jose Guevara-Coto, Ram Yogendra, Edgar B. Francisco, Emily Long, Amruta Pise, Hallison Rodrigues, Purvi Parikh, Javier Mora, and Rodrigo A. Mora-Rodríguez. 2021. “Immune-Based Prediction of COVID-19 Severity and Chronicity Decoded Using Machine Learning.” Frontiers in Immunology 12 (June): 700782.

[16] Li, Mengyu, Chris Ho Ching Yeung, and C. Mary Schooling. 2021. “Circulating Cytokines and Coronavirus Disease: A Bi-Directional Mendelian Randomization Study.” Frontiers in Genetics 12 (June): 680646.

[17] El Tabaa, Manar Mohammed, and Maram Mohammed El Tabaa. 2020. “New Putative Insights into Neprilysin (NEP)-Dependent Pharmacotherapeutic Role of Roflumilast in Treating COVID-19.” European Journal of Pharmacology 889 (173615): 173615.

[18] Mohammed El Tabaa, Manar, and Maram Mohammed El Tabaa. 2020. “Targeting Neprilysin (NEP) Pathways: A Potential New Hope to Defeat COVID-19 Ghost.” Biochemical Pharmacology 178 (114057): 114057.

[19] Bellis, Alessandro, Ciro Mauro, Emanuele Barbato, Bruno Trimarco, and Carmine Morisco. 2020. “The Rationale for Angiotensin Receptor Neprilysin Inhibitors in a Multi-Targeted Therapeutic Approach to COVID-19.” International Journal of Molecular Sciences 21 (22): 8612.

[20] Zolfaghari Emameh, Reza, Reza Falak, and Elham Bahreini. 2020. “Application of System Biology to Explore the Association of Neprilysin, Angiotensin-Converting Enzyme 2 (ACE2), and Carbonic Anhydrase (CA) in Pathogenesis of SARS-CoV-2.” Biological Procedures Online 22 (1): 11.

[21] Rex, Devasahayam Arokia Balaya, Sumaithangi Thattai Arun Kumar, Prashant Kumar Modi, and Thottethodi Subrahmanya Keshava Prasad. 2021. “Broadening COVID-19 Interventions to Drug Innovation: Neprilysin Pathway as a Friend, Foe, or Promising Molecular Target?” Omics: A Journal of Integrative Biology 25 (7): 408–16.

[22] Li, Linlin, Mingxiang Huang, Jianshan Shen, Yao Wang, Rui Wang, Cai Yuan, Longguang Jiang, and Mingdong Huang. 2021. “Serum Levels of Soluble Platelet Endothelial Cell Adhesion Molecule 1 in COVID-19 Patients Are Associated With Disease Severity.” The Journal of Infectious Diseases.

[23] Seery, Vanesa, Silvina C. Raiden, Silvia C. Algieri, Nicolás A. Grisolía, Daniela Filippo, Norberto De Carli, Sandra Di Lalla, et al. 2021. “Blood Neutrophils from Children with COVID-19 Exhibit Both Inflammatory and Anti-Inflammatory Markers.” EBioMedicine 67 (103357): 103357.

[24] Costanzo, Luca, Francesco Paolo Palumbo, Giorgio Ardita, Pier Luigi Antignani, Enrico Arosio, Giacomo Failla, and Italian Society for Vascular Investigation and the Italian Society of Vascular Medicine. 2020. “Coagulopathy, Thromboembolic Complications, and the Use of Heparin in COVID-19 Pneumonia.” Journal of Vascular Surgery. Venous and Lymphatic Disorders 8 (5): 711–16.

[25] Smadja, David M., Coralie L. Guerin, Richard Chocron, Nader Yatim, Jeremy Boussier, Nicolas Gendron, Lina Khider, et al. 2020. “Angiopoietin-2 as a Marker of Endothelial Activation Is a Good Predictor Factor for Intensive Care Unit Admission of COVID-19 Patients.” Angiogenesis 23 (4): 611–20.

[26] Spadaro, Savino, Alberto Fogagnolo, Gianluca Campo, Ottavio Zucchetti, Marco Verri, Irene Ottaviani, Tanushree Tunstall, et al. 2021. “Markers of Endothelial and Epithelial Pulmonary Injury in Mechanically Ventilated COVID-19 ICU Patients.” Critical Care (London, England) 25 (1): 74.

[27] Birnhuber, Anna, Elisabeth Fliesser, Gregor Gorkiewicz, Martin Zacharias, Benjamin Seeliger, Sascha David, Tobias Welte, et al. 2021. “Between Inflammation and Thrombosis - Endothelial Cells in COVID-19.” The European Respiratory Journal: Official Journal of the European Society for Clinical Respiratory Physiology, May. https://doi.org/10.1183/13993003.00377-2021.

[28] Leng, Ling, Ruiyuan Cao, Jie Ma, Danlei Mou, Yunping Zhu, Wei Li, Luye Lv, et al. 2020. “Pathological Features of COVID-19-Associated Lung Injury: A Preliminary Proteomics Report Based on Clinical Samples.” Signal Transduction and Targeted Therapy 5 (1): 240.

[29] Wu, Huaying, Ke Gong, You Qin, Zhiying Yuan, Shuaishuai Xia, Shiying Zhang, Jingjing Yang, Ping Yang, Liang Li, and Mengzhou Xie. 2021. “In Silico Analysis of the Potential Mechanism of a Preventive Chinese Medicine Formula on Coronavirus Disease 2019.” Journal of Ethnopharmacology 275 (114098): 114098.

[30] Chen, Liang, and Sika Zheng. 2020. “Understand Variability of COVID-19 through Population and Tissue Variations in Expression of SARS-CoV-2 Host Genes.” Informatics in Medicine Unlocked 21 (100443): 100443.

[31] He, Bing, and Lana Garmire. 2020. “Prediction of Repurposed Drugs for Treating Lung Injury in COVID-19.” F1000Research 9 (June): 609.

[32] Hamad, Mosab Nouraldein Mohammed. 2020. “Blood Group Type, Intercellular Adhesion Molecule-1 (ICAM-1) and Angiotensin-2 Im-Pact on COVID. 19 Outcomes.” EC Endocrinology and Metabolic Research 5. <https://www.researchgate.net/profile/Mosab-Nouraldein-Mohammed-Hamad/publication/344898824_Blood_Group_Type_Intercellular_Adhesion_Molecule-1_ICAM-1_and_Angiotensin-2_Impact_on_COVID19_Outcomes/links/5f97e793299bf1b53e4992ac/Blood-Group-Type-Intercellular-Adhesion-Molecule-1-ICAM-1-and-Angiotensin-2-Impact-on-COVID19-Outcomes.pdf>.

[33] Tong, Ming, Yu Jiang, Da Xia, Ying Xiong, Qing Zheng, Fang Chen, Lianhong Zou, Wen Xiao, and Yimin Zhu. 2020. “Elevated Expression of Serum Endothelial Cell Adhesion Molecules in COVID-19 Patients.” The Journal of Infectious Diseases 222 (6): 894–98.

[34] Nagashima, Seigo, Monalisa Castilho Mendes, Ana Paula Camargo Martins, Nícolas Henrique Borges, Thiago Mateus Godoy, Anna Flavia Ribeiro dos Santos Miggiolaro, Felipe da Silva Dezidério, Cleber Machado-Souza, and Lucia de Noronha. 2020. “Endothelial Dysfunction and Thrombosis in Patients with COVID-19—Brief Report.” Arteriosclerosis, Thrombosis, and Vascular Biology 40 (10): 2404–7.
