# Supplementary material for: Proteome-wide Mendelian randomization identifies causal links between blood proteins and severe COVID-19
Source: PLoS Genet. 2022 Mar 3;18(3):e1010042. doi: 10.1371/journal.pgen.1010042 (PMC8893330; doi:10.1371/journal.pgen.1010042)
Supplement: S4 Fig — (DOCX) [file pgen.1010042.s015.docx]

# S4 Fig. Effect plots of blood markers associated with a decrease in risk of respiratory support/death as a result of COVID-19


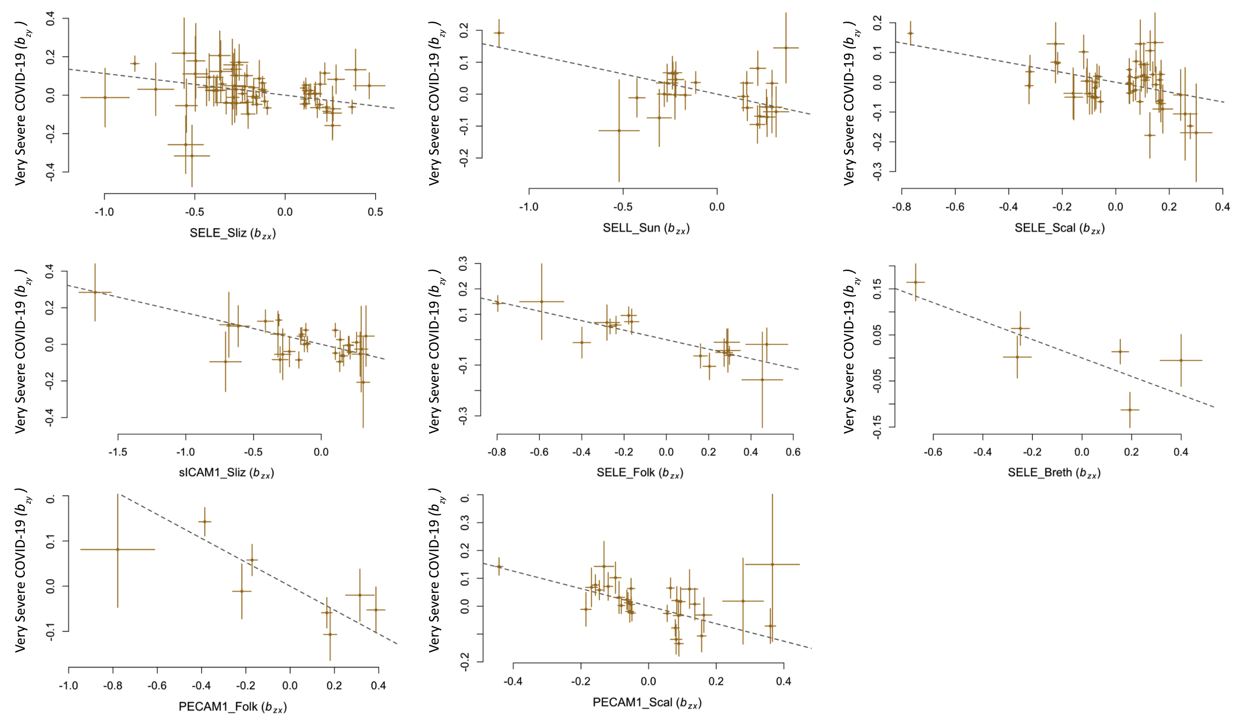


This figure illustrates the SNP effect relationship between significant blood markers (exposure) and an decreased risk of respiratory support/death as a result of COVID-19 (outcome). SNP effects associated with blood markers are displayed on the x-axis and SNP effects associated with respiratory support/death as a result of COVID-19 are displayed on the y-axis. Each point represents the beta coefficient of these two traits. The horizontal line on each point represents the standard error associated with the exposure and the vertical line on each point represents the standard error associated with the outcome. The dotted line represents the regression line between these two traits.
