# Supplementary material for: Proteome-wide Mendelian randomization identifies causal links between blood proteins and severe COVID-19
Source: PLoS Genet. 2022 Mar 3;18(3):e1010042. doi: 10.1371/journal.pgen.1010042 (PMC8893330; doi:10.1371/journal.pgen.1010042)
Supplement: S5 Fig — (DOCX) [file pgen.1010042.s016.docx]

## S5 Fig. Plot indicating the number of heterogeneous SNP instruments on chromosome 9 from biomarkers identified in the hospitalization from COVID-19 GWAS.


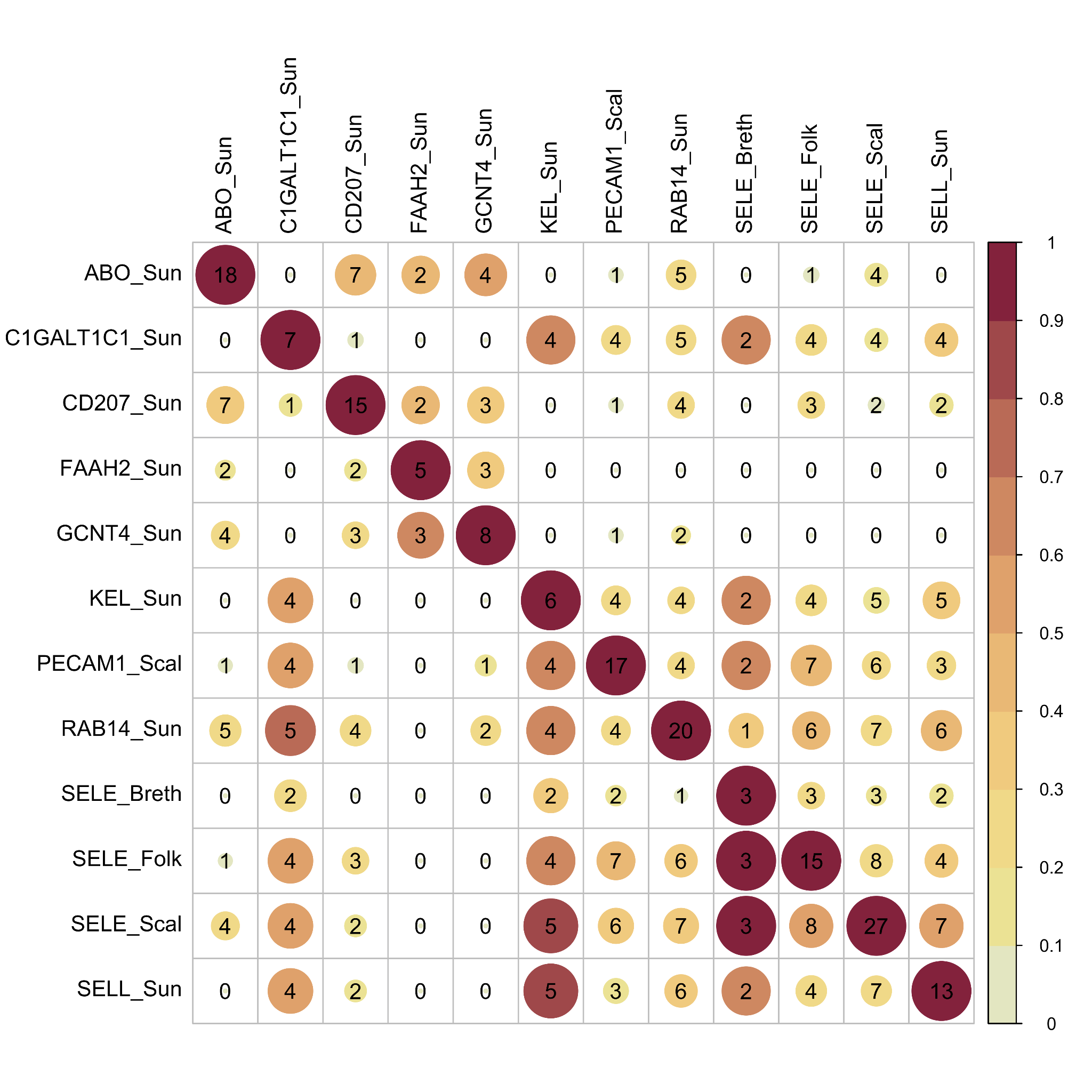


This plot displays the number of SNPs in high linkage disequilibrium (r2>0.6) used as instruments in blood proteins associated with higher/lower odds of COVID-19 hospitalization, located on chromosome 9 between different blood markers. The diagonal indicates the total number of instruments for each marker on chromosome 9. The colour and size of the circles indicates which proportion of SNPs shared between the marker in each row and the corresponding column marker. ABO = ABO system transferase; ATP2A3 = ATPase Sarcoplasmic/Endoplasmic Reticulum Ca2+ Transporting 3; C1GALT1C1 = C1GALT1 specific chaperone 1; CD207 = langerin; FAAH2 = Fatty Acid Amide Hydrolase 2; GCNT4 = glucosaminyl (N-Acetyl) transferase 4; KEL = Kell Metallo-Endopeptidase (Kell Blood Group); LCTL = Lactase-like protein; PECAM1 = platelet endothelial cell adhesion molecule; RAB14 = ras-related protein rab-14; SELE = E-selectin; SELL = L-selectin; SFTPD = Surfactant Protein D.
