# Supplementary material for: Proteome-wide Mendelian randomization identifies causal links between blood proteins and severe COVID-19
Source: PLoS Genet. 2022 Mar 3;18(3):e1010042. doi: 10.1371/journal.pgen.1010042 (PMC8893330; doi:10.1371/journal.pgen.1010042)
Supplement: Attachment — Submitted filename: PlosGen_Response Letter.docx [file pgen.1010042.s019.docx]

Vienna, November 20^th^, 2021

**Subject**:	**Response letter**

*Proteome-wide Mendelian randomization identifies causal links between blood proteins and severe COVID-19, Submission ID: PGENETICS-D-21-01126*

Dear Dr. Chris Cotsapas:

We thank you for handling our manuscript, as well as the reviewers for their thorough review, helpful critique, and encouraging words.

We have addressed all reviewer’s comments and revised the manuscript accordingly. Please find our responses to specific comments below.

To note, we have fully responded to all comments on a point-by-point basis, adding our response below each of the “Comments to Author”. Please find the reviewer’s comments in bold, our answer in plain text, and our additions to the manuscript in italics.

Given that results from the new analyses, as requested by reviewers, have meant that major parts of the manuscript have been rewritten, we have not tracked all changes but have rather highlighted parts of the manuscript that have changed considerably.

We confirm that all authors concur with the resubmission. The material in this manuscript constitutes original research, has not been previously published and has not been submitted for publication elsewhere.

Hoping that you will consider our improved article for publication in PLOS Genetics, I remain

Yours sincerely,

Vincent Millischer, MD, PhD

-----------------------------------------------------------------------------------

Department of Psychiatry and Psychotherapy,

Medical University of Vienna, Austria &

Department of Molecular Medicine and Surgery,

Karolinska Institutet, Stockholm, Sweden

**Comments to the Authors:**

**Reviewer #1:**

**1. Please show scatter plots for the effect size estimates of your instrumental variables on the protein level and on COVID risk, respectively. These are helpful to evaluate whether the correlation is uniform, with all variants affecting protein level also affecting risk, or whether it is driven by just one or two shared variants.**

**Response:** We thank the reviewer for this suggestion. Scatter plots are helpful to evaluate the findings and we have included scatter plots in Supplementary Figures 1-4.

**2. Please report the effect, if any, of variants that are in the cis region of the protein that they encode. Such variants make relatively strong instrumental variables, because their effects are unlikely to be mediated by a confounder in trans.**

**Response:** We performed an additional sensitivity analysis including cis-variants where possible. We have updated our manuscript as follows:

Methods:

*Furthermore, when possible, we performed GSMR using only variants in the cis-region of the gene encoding the blood marker (defined as variants either within a gene, up to 1 Mb proximal to the start of the gene, or up to 1 Mb distal to the end of the gene). Gene information was obtained from Ensembl (Howe et al. 2021) using the biomaRt library (Durinck et al. 2009), SNP information was obtained from NCBI dbSNP (Sherry et al. 2001) using the rsnps library (“Get ‘SNP’ (’Single-Nucleotide' ‘Polymorphism’) Data on the Web [R Package Rsnps Version 0.4.0]” 2020).*

Results:

*In order to establish whether the significant associations identified in our study were being driven by cis-regulatory variants, we identified cis-SNPs from all significant blood markers and performed Mendelian randomization analyses using only these SNPs with the respective COVID-19 GWASs. The results show that ABO_Sun and SFTPD_Breth cis-SNPs are significantly associated with hospitalization as a result of COVID-19, and ABO_Sun and sICAM-1_Sliz cis-SNPs are significnatly associated with severe COVID-19. All other significant associations are deemed to be driven by trans-SNPs (Supplementary Material, Section 6).*

**3. Please check if any of your instrumental variables are pleiotropically associated with BMI, which would be an obvious confounder. (It may affect both protein levels and severe COVID risk).**

**Response:** We have added a sensitivity analysis to better understand the relationship between BMI, COVID-19 severity, and protein levels. We adapted the manuscript as follows:

Methods:

*With BMI being associated with both COVID-19 severity and the levels of many inflammatory proteins* [*(Palmos et al. 2020; Greenberg and Obin 2006; Soeroto et al. 2020)*](https://paperpile.com/c/T3d3Zr/K9rG+tJlh+UFGe)*, we also performed GSMR with BMI both as exposure and outcome for all significant proteins.*

Results:

*Given that high BMI has been robustly associated with both inflammatory cytokine levels in the blood and an increased risk of severe COVID-19* [*(Palmos et al. 2020; Greenberg and Obin 2006; Soeroto et al. 2020)*](https://paperpile.com/c/T3d3Zr/K9rG+tJlh+UFGe)*, we performed bidirectional Mendelian randomization analyses between all significant blood markers and BMI, using the largest BMI GWAS* [*(Yengo et al. 2018)*](https://paperpile.com/c/T3d3Zr/Sg11)*. Our results indicate that genetic susceptibility for higher BMI is associated with higher levels of SELE_Scal, C1GALT1C1_Sun, SELE_Folk, KEL_Sun, SELL_Sun, RAB14_Sun and SFTPD_Breth. In addition, the genetic susceptibility for higher levels of LCTL_Sun, SELE_Sliz, SFTPD_Breth, PECAM-1_Scal and RAB14_Sun is associated with higher BMI (see* ***Supplementary Material****, Section 7 for a full results). Note that some GWAS had controlled for BMI, whereas others, the majority of which show a significant association with BMI, did not (see* ***Supplementary Material****, Section 1).*

Discussion:
*Sixth, our sensitivity analyses demonstrate that a genetic predisposition to high BMI is significantly associated with some of our blood markers. High BMI is also associated with severe COVID-19* [*(Soeroto et al. 2020)*](https://paperpile.com/c/T3d3Zr/UFGe)*, suggesting that it could be the driving factor behind increased blood protein levels. However, some blood protein GWASs controlled for BMI which should reduce the potential confounding (****Supplementary Material****, Section 1).*

Supplementary Materials:
*We have highlighted the studies controlling for BMI (Supplementary Table 1) and have added a Supplementary Table 7: Blood marker associations with BMI.*

**Also, please check which of your results change if the HLA region (which has very long-range LD and unusually strong associations with numerous phenotypes) is excluded from the analysis.**

**Response:** With the updated analyses using the new GWASs for both “severe COVID-19 phenotypes: hospitalization and respiratory support/death, no SNP in the HLA region was used as an instrument. Therefore, the suggestion does not apply to our analysis anymore.

**I’ll just highlight one particular result as needing more attention and discussion: the ABO finding seems concordant with early reports of a blood-type association, but my impression was that recent evidence has shown this to be a false positive, possibly due to stratification. Is the ABO association supported by any variants outside of the disputed locus?**

**Response:** To our knowledge, the evidence supporting an association between the ABO system and COVID-19 severity is quite strong, supported by both meta-analyses (cf. <https://doi.org/10.1016/j.meegid.2020.104485>) and large scale cohort studies (cf. <https://doi.org/10.7326/M20-4511>). In our analysis, the ABO locus survived the cis-variant sensitivity analysis which supports this finding.

**Reviewer #2:**

**Major comments**

**1. Poor choice of COVID GWAS**

**a) The primary phenotype is taken from the host genetic initiative release 5, which makes sense as these were presented in the Nature paper (reference 7) published earlier this year. From this paper, the authors have selected the GWAS results: “A2_ALL_leave_UKBB: Very severe respiratory confirmed covid (5,870) vs. population (1,155,203)” incorrectly calling it the European only sample. I agree it makes sense to use the European sample, they should have selected: “A2_ALL_eur: Very severe respiratory confirmed covid (5,101) vs. population (1,383,241)”**

**b) The authors have chosen to label this phenotype “hospitalized-COVID-19 GWAS” even though Ganna et al called this phenotype “critically ill COVID-19+” ; Ganna et al used the name “Hospitalised COVID” for a less severe phenotype 13K case 2M controls (or just Europeans B2_ALL_eur: (9,986) vs. population (1,877,672)). I don’t think it is helpful to mix up names in this way. No doubt the reason for this was because the authors tried to select a what they thought was a more phenotype selecting from release 4, “Very severe respiratory confirmed covid vs. not hospitalized covid” 269 cases vs 688 controls (A1_ALL). It is not clear why this sample was chosen it was very small, has no GWS SNPs, and while I don’t think this analysis is needed (the very large population control makes more sense) release 5 has much bigger samples for this phenotype 4,829 and 11,816 (B1_ALL_eur).**

**Response:** We thank the reviewer for this valuable comment and have repeated our study with the COVID-19-GWASs that better fit the aims of our analyses and the ethnic background of the blood marker GWASs. As the European only samples were not available for release 6, we performed analyses on the GWASs of release 5 (i.e. B2_ALL_eur and A2_ALL_eur). We have also clarified the phenotypes, using “hospitalization” and “need for respiratory support/death”. The B2_ALL_eur GWAS was used instead of B1_All_eur to maximise power to detect meaningful associations. In the manuscript, we have adapted the methods section as follows:

Methods:

*In order to capture increased risk of hospitalization as a result of severe COVID-19 we downloaded the COVID-19 Host Genome Initiative GWAS meta-analysis* [*(COVID-19 Host Genetics Initiative 2021)*](https://paperpile.com/c/T3d3Zr/N2DzO) *of “Hospitalized covid vs. population”, European ancestry (B2_ALL_eur, release 5, January 2021;* [*https://www.covid19hg.org/results/r5/*](https://www.covid19hg.org/results/r5/)*). Cases were defined as SARS-CoV-2 infected individuals who required hospitalization due to COVID-19 related symptoms. Controls were defined as non-cases, i.e. the population. The European sample consisted of 9,986 cases and 1,877,672 controls. In our study, we refer to this GWAS as the hospitalization-COVID-19 GWAS.*

*In order to capture increased risk of very severe respiratory COVID-19, including respiratory support and death, we downloaded the COVID-19 Host Genome Initiative GWAS meta-analysis* [*(COVID-19 Host Genetics Initiative 2021)*](https://paperpile.com/c/T3d3Zr/N2DzO) *of “very severe respiratory confirmed covid vs. population”, European ancestry (A2_ALL_eur, release 5, January 2021;* [*www.covid19hg.org/results/*](https://www.covid19hg.org/results/)*r5). Cases were defined as SARS-CoV-2 infected individuals who were admitted to hospital, had COVID-19 as the primary reason for admission, and had died or needed respiratory support (i.e., intubation, continuous positive airway pressure or bilevel positive airway pressure). Controls were defined as non-cases, i.e. the population* [*(COVID-19 Host Genetics Initiative 2021)*](https://paperpile.com/c/T3d3Zr/N2DzO)*. The European sample consisted of 5,101 cases and 1,383,241 controls. In this study we refer to this GWAS as the respiratory support/death-COVID-19 GWAS.*

**2. Non-reproducibility of protein GWAS.**

**From what is written or available in the supplement I am not sure if others could repeat the analysis.**

**a) Supplementary Table 1 lists 10 studies but five of these use the OLINK panel – were these meta-analysed?**

**Response:** As we have not meta-analysed the studies, we have clarified this point in our manuscript as follows:

Methods:

*Note that all protein measurements from all studies described above were included in our analyses, meaning that some proteins were analysed more than once.*

Results:

*Our results show for some proteins robust associations with the same proteins twice, as they were measured twice in independent GWASs, serving as direct replication. In order to easily identify these proteins, we added a suffix of the study name to the protein.*

Discussion:

*We did, however, estimate associations for some proteins twice as their GWAS originated from independent GWASs. These results displayed the same effects, rendering them a direct replication, increasing the validity of our findings.*

**b) “We tested 4,366 associations with hospitalized-COVID-19 as the exposure and the blood proteins as outcome”. So is 4,366 the number of proteins or the number of SNPs? Suggest you provide a supplementary data file listing the SNPs used as instruments for each protein.**

**Response:** To make our analysis more reproducible, we have included the full list of SNPs used as instruments for all proteins that were significantly associated in our analyses in the supplementary data. We have also added a column indicating the link and origin of the summary statistics used in the analyses in Supplementary Table 1.

**Minor comments:**

**1) Referring to the differences in GWAS results severe vs hospitalised a statement is made “The findings suggest that one set of individual genetic variants may be responsible for hospitalization, and once hospitalized, another set of genetic variants may be responsible for respiratory failure and death.” This statement and elsewhere provides a feeling that the researchers have not fully engaged with the severe-COVID19 phenotype. This statement makes these findings sound novel, but this difference is well known clinically. For example, the GENOMICC GWAS (Pairo-Castineira et al) said “In the UK, the group of patients admitted to critical care is relatively homogeneous, with profound hypoxaemic respiratory failure being the archetypal presentation “ and “Patients admitted to intensive care units in the UK during the first wave of COVID-19 were, on average, younger and less burdened by comorbid illnesses than the hospitalized population” – citing Docherty et al.**

**Response:** The revised analyses with new summary statistics, which are better adapted regarding the sample size and the ethnicity, do not point to major differences between the two phenotypes. We have adapted our manuscript accordingly. Note that we do not focus on any specific clinical presentations of COVID-19, but are rather investigating the role of circulating blood markers in their putative causal association with two broad COVID-19 phenotypes. Our findings support previous clinical studies in terms of translational findings, but also identify potentially novel biological mechanisms. We have made a note of previously reported distinctions in clinical manifestations (hospitalized vs critically ill) of COVID-19:

Introduction:

*COVID-19 pathology encompasses a wide spectrum of clinical manifestations from asymptomatic, mild, moderate, to 15% being severe infections (Blanco-Melo et al. 2020; García 2020, Docherty et al. 2020).*

**2) What are the units of the Beta and odds ratios? Are the units the same for all analyses?**

**Response:** Thank you for pointing this out. We have looked into the units used in the original protein GWAS. Several studies use different units of measurement, but most studies, including the largest studies which give the highest number of significant associations, use a standardized score. Therefore, we feel reporting them as an increase per standard deviation is most appropriate. We have mentioned this in our results.

Results:

*In addition, note that units of protein measurement differed in studies, with some studies using standardized units (see studies in* ***Supplementary Table 1****). Thus, we will report our findings per standard deviation increase.*

**3) gwasrapidd not gwasrapid**

**Response:** We have corrected it in the manuscript.

**4) Statements such as “the genetic liability for exposure X is causal for outcome Y” are made on several occasions. These statements are commonly found in MR publications but in my opinion miss the point about MR. The point is that using genetic instruments and under the assumptions of MR results are consistent with exposure X being causal for outcome Y. There is a subtle difference in meaning here**

**Response:** We have changed the wording throughout the manuscript.

**5) In this study, the 1,000 Genomes dataset was the reference dataset (18). Do you mean the European ancestry samples to match the EUR ancestry GWAS?**

**Response:** We have clarified this point in the method section:

*In this study, the European 1,000 Genomes dataset was used as the reference dataset (Wood et al. 2013).*

**6) To get sufficient SNP instruments to conduct MR, the significance threshold was set at p < 5 x 10-6. This decision is justified through reference to 5 papers Ref 26-30, which includes one paper by the authors, and three rather old papers. Just because other papers have passed through peer review it does not justify the approach, this must be justified by a methodological paper. Generally, I believe use of weak instruments is not supported.**

**Response:**

We thank the reviewer for raising this important issue. Due to small sample sizes currently available in GWAS measuring protein levels, a reduced p-value threshold is needed in order to obtain SNP instruments to be used in MR analyses. Although we agree that this could introduce a degree of error, we believe our results highlight the validity of our SNP instruments by having significant blood markers, measured in different independent studies, with the same direction of effect, comparable effect sizes with overlapping confidence intervals. In addition, a number of significant markers have been previously reported in other clinical, observational and genetic studies, suggesting that we are indeed picking up valid signals. Finally, we have added tests for the F-statistic and the I-squared statistic, which show that our genetic instruments are indeed valid. These have been included in the manuscript.

Methods:

*...we additionally calculated F-statistics and I-squared statistics to transparently present the strength of our instruments (****Supplementary Table 9****). We needed to take this analytical step because GWAS of blood proteins with more statistical power are at time of your study not available.*

Results:

*Given that we are using a reduced p-value threshold to identify SNPs to be used as our genetic instruments, we ran F-statistic and I-squared statistic calculations on all of our significantly associated proteins. We found that with the exception of ATP2A3, all F-statistics were larger than 20 and all I-squared statistics were larger than 0.9, suggesting strong genetic instruments for use in analyses* [*(Bowden et al. 2016; Zhao et al. 2018; Bowden et al. 2019)*](https://paperpile.com/c/T3d3Zr/ndI1+4Qw2+ZYmk)*. See* ***Supplementary Material****, Section 9 full results of these analyses.*

Discussion:
*First, although we required confirmation of our findings by several Mendelian randomization methods, we set the p-value threshold for selecting genetic variants as our instruments at genome-wide suggestive significance (p < 5 x 10^-6^) to identify enough SNP instruments for each protein to run Mendelian randomization analyses. Some genetic variants may therefore be false positive associations with protein levels. This procedure is common in Mendelian randomization analysis* [*(Hübel et al. 2019; Holmes et al. 2015; Censin et al. 2017; Wootton et al. 2021; Gariepy, Nitka, and Schmitz 2010)*](https://paperpile.com/c/T3d3Zr/GbEhS+RYtDH+yHmd2+yLfCW+Ft10N)*. Additionally, we report F-statistics and I-squared statistics per SNP instrument (****Supplementary Table 9****) so that the quality of our instruments is transparent. This step was necessary as the available GWASs of the blood proteins are still of limited sample size; and therefore statistical power.*
