# Supplementary material for: Proteome-wide Mendelian randomization identifies causal links between blood proteins and severe COVID-19
Source: PLoS Genet. 2022 Mar 3;18(3):e1010042. doi: 10.1371/journal.pgen.1010042 (PMC8893330; doi:10.1371/journal.pgen.1010042)
Supplement: Attachment — Submitted filename: PlosGen_Response Letter_resubmission_2_211219.docx [file pgen.1010042.s020.docx]

Vienna, January 6^th^, 2021

**Subject**:	**Response letter**

*Proteome-wide Mendelian randomization identifies causal links between blood proteins and severe COVID-19, Submission ID: PGENETICS-D-21-01126*

Dear Dr. Chris Cotsapas:

We thank you for handling our manuscript, as well as the reviewers reviewing our manuscript a second time.

We have addressed the editor’s as well as the reviewer’s comments and revised the manuscript accordingly. Please, find our responses to specific comments below.

To note, we have fully responded to all comments on a point-by-point basis, adding our response below each of the “Comments to Author”. Please find the reviewer’s comments in bold, our answer in plain text, and our additions to the manuscript in italics. We tracked our changes in a new version.

We also provide a Striking Image designed using BioRender and hope it will be considered for featuring on the website.

We confirm that all authors concur with the resubmission. The material in this manuscript constitutes original research, has not been previously published and has not been submitted for publication elsewhere.

Hoping that you will consider our improved article for publication in PLOS Genetics, I remain

Yours sincerely,

Vincent Millischer, MD, PhD

-----------------------------------------------------------------------------------

Department of Psychiatry and Psychotherapy,

Medical University of Vienna, Austria &

Department of Molecular Medicine and Surgery,

Karolinska Institutet, Stockholm, Sweden

**Comments to the Authors:**

**Editorial comments:**

1. **Please be aware that our** [**data availability policy**](http://journals.plos.org/plosgenetics/s/data-availability) **requires that all numerical data underlying graphs or summary statistics are included with the submission, and you will need to provide this upon resubmission if not already present.**

**In addition, we do not permit the inclusion of phrases such as "data not shown" or "unpublished results" in manuscripts. All points should be backed up by data provided with the submission.**

**Response:** We have provided all data in the Supplementary Data Excel sheet, “PWMR_PG_Resubmission_Full_Results_Tables.xlsx”

2. **To enhance the reproducibility of your results, we recommend that you deposit your laboratory protocols in protocols.io, where a protocol can be assigned its own identifier (DOI) such that it can be cited independently in the future. Additionally, PLOS ONE offers an option to publish peer-reviewed clinical study protocols. Read more information on sharing protocols at** [**https://plos.org/protocols?utm_medium=editorial-email&utm_source=authorletters&utm_campaign=protocols**](https://plos.org/protocols?utm_medium=editorial-email&utm_source=authorletters&utm_campaign=protocols)

**Response:** We thank the editors for this recommendation. We are now sharing our analyses scripts on GitHub. We have updated the data availability section as follows:

*The links to the summary statistics can be found in Supplementary Table 1. Analyses scripts are publicly available under https://github.com/tnggroup/PWMR_Covid19.*

3. **Please review your reference list to ensure that it is complete and correct. If you have cited papers that have been retracted, please include the rationale for doing so in the manuscript text, or remove these references and replace them with relevant current references. Any changes to the reference list should be mentioned in the rebuttal letter that accompanies your revised manuscript. If you need to cite a retracted article, indicate the article’s retracted status in the References list and also include a citation and full reference for the retraction notice.**

Response: We have reviewed our reference list and corrected as follows.

1) We have corrected following references:

COVID-19 Host Genetics Initiative. 2021. “Mapping the Human Genetic Architecture of COVID-19.” Nature 600 (7889): 472–77.

NHS, “Blood Types.” [online], Accessed November 11, 2021. <https://www.blood.co.uk/why-give-blood/blood-types/>.

Gustavsen J, Rüeger S, Chamberlain S, Ushey K, Zhu H. 2021. rsnps: Get 'SNP' ('Single-Nucleotide' 'Polymorphism') Data on the Web. https://docs.ropensci.org/rsnps/, https://github.com/ropensci/rsnps/.

2) Following reference has now been published, we have therefore replaced the reference to the preprint to following:

Zhao, Qingyuan, Jingshu Wang, Gibran Hemani, Jack Bowden, and Dylan S. Small. 2020. “Statistical Inference in Two-Sample Summary-Data Mendelian Randomization Using Robust Adjusted Profile Score.” Annals of Statistics 48 (3): 1742–69.

3) We have added the issue and volume in following reference:

Thépaut, Michel, Joanna Luczkowiak, Corinne Vivès, Nuria Labiod, Isabelle Bally, Fátima Lasala, Yasmina Grimoire, et al. 2021. “DC/L-SIGN Recognition of Spike Glycoprotein Promotes SARS-CoV-2 Trans-Infection and Can Be Inhibited by a Glycomimetic Antagonist.” PLoS Pathogens 17 (5): e1009576.

Zhao, Jiao, Yan Yang, Hanping Huang, Dong Li, Dongfeng Gu, Xiangfeng Lu, Zheng Zhang, et al. 2020. “Relationship between the ABO Blood Group and the COVID-19 Susceptibility.” Clinical Infectious Diseases: An Official Publication of the Infectious Diseases Society of America 73 (2): 328–31.

4) We have clarified that following reference is a thesis:

Martin, J. 2009. [Thesis]. “Role of Fatty Acid Binding Proteins and FAAH-2 in Endocannabinoid Uptake and Inactivation.” Edited by Dale G. Deutsch. Ann Arbor, United States: State University of New York at Stony Brook. https://www.proquest.com/dissertations-theses/role-fatty-acid-binding-proteins-faah-2/docview/305091515/se-2.

**Reviewer #1:**

1. **In Supplementary Figures 1-4, the authors show scatterplots for their significant associations. Most of these look quite good, better than a lot of MR analyses, indicating that most protein-associated SNPs have proportional effects on hospitalization risk. For example slCAM1, which also passed the cis-variant sensitivity test, looks like it is supported by a large number of instruments with consistent effects. In contrast the ABO plot looks fairly poor, with many ABO-associated SNPs having discordant effects on the outcome. I think these results merit some discussion in the main text. Also, please fix the figure labels in Supp Fig 1, some of which are cut off.**

**Response:** We thank the reviewer for their review of our re-submission. We have corrected the missing labels in Supplementary Fig 1. Furthermore, we have added following part to the discussion:

Discussion:

*In addition, we note that for most associations (such as between FAAH2 and hospitalized COVID-19, or sICAM1 and severe COVID-19) we observe a clear linear relationship (Supplementary Figures 1–4). However, some associations, such as between ABO and hospitalized COVID-19, or GCNT4 and severe COVID-19, display SNP effects between the exposure and outcome with very high standard errors. While reducing the validity of these SNPs as SNPs with robust predictive power, the joint effect over all SNPs remains significant.*

2. **The BMI analyses do seem to indicate that confounding due to BMI is potentially quite important. Please add some discussion about whether the direction of effect that you observed was consistent with potential confounding – i.e., when BMI-associated variants are associated with increased protein levels, are those the same proteins for which increased protein-increasing variants are associated with increased COVID risk?**

**Response:** To clarify the relationships, we have performed an MR between BMI and COVID, showing that BMI is strongly associated with Covid-19 risk. The data are shown in the supplements (*Supplementary Table 8*). We also commented on the question in the discussion.

*Results:*

*After validating this relationship by performing Mendelian randomization analysis between BMI and the two COVID-19 outcomes (Supplementary Table 8), we performed bidirectional Mendelian randomization analyses between all significant blood markers and BMI, using the largest publicly available BMI GWAS (Yengo et al. 2018).*

*Discussion:*

*High BMI is also associated with severe COVID-19 (Soeroto et al. 2020, Supplementary Table 8), suggesting that BMI may drive risk of severe forms of COVID-19 and the change in blood protein levels, potentially confounding our results. However, our Mendelian randomization analyses showed that higher BMI is causal for higher levels of the COVID-19 protective proteins SELE, KEL, SELL, and causal for lower levels of the COVID-19 risk increasing proteins C1GALT and RAB14. Our sensitivity analysis therefore shows that BMI influences these blood markers in opposite directions than would be concurrent with confounding effects. This indicates the effects of these proteins are independent of BMI.*

3. **Regarding the cis analysis, you might report that the total number of proteins that had testable cis variants (which was smaller than I expected, if I understand correctly); initially I got the impression that most proteins failed this sensitivity analyses, when it was actually not applicable. Please add a caption to Supplementary Table 6, and cite it in the text as a Table not as a Note.**

Response: We clarified our sensitivity analysis. Only few exposure-outcome pairs were based on at least one cis-SNP. We added the following in the results section:

*Out of the 28 exposure-outcome pairs, seven (25%) where based on at least one cis-SNP and could therefore be analysed.*

We changed the title of and reference to Supplementary Table 6 and have added a caption:

*This table displays associations between blood proteins that have a significant effect (exposure) in COVID-19 phenotypes (outcomes) in our analysis, using only cis-SNPs as genetic instruments. We did not identify cis-SNPs for other blood proteins.*

**Reviewer #2:**

1. The authors have addressed my comments, except Point 4, I leave in the hands of the editor.
   Point 4 (first revision): Statements such as “the genetic liability for exposure X is causal for outcome Y” are made on several occasions. These statements are commonly found in MR publications but in my opinion miss the point about MR. The point is that using genetic instruments and under the assumptions of MR results are consistent with exposure X being causal for outcome Y. There is a subtle difference in meaning here.

Response: We apologise for the misunderstanding in the previous round of reviews. We changed the wording throughout the manuscript, using the suggested formulation. All changes can be found in the tracked version, and have been formulated like following example:

*After multiple testing correction (pFDR = 0.05), using genetic instruments and under the assumptions of Mendelian randomization, our results were consistent with eight blood markers being causally associated with a statistically significantly decreased risk of need for respiratory support/death due to COVID-19.*
